# Vascular Smooth Muscle Cell Plasticity and Autophagy in Dissecting Aortic Aneurysms

**DOI:** 10.1161/ATVBAHA.118.311727

**Published:** 2019-04-04

**Authors:** Marc Clément, Joel Chappell, Juliette Raffort, Fabien Lareyre, Marie Vandestienne, Annabel L. Taylor, Alison Finigan, James Harrison, Martin R. Bennett, Patrick Bruneval, Soraya Taleb, Helle F. Jørgensen, Ziad Mallat

**Affiliations:** 1 From the Division of Cardiovascular Medicine, University of Cambridge, United Kingdom (M.C., J.C., J.R., F.L., A.L.T., A.F., J.H., M.R.B., H.F.J., Z.M.); 2 Institut National de la Santé et de la Recherche Médicale, Universite Paris-Descartes, Paris Cardiovascular Research Center, and Université Paris-Descartes, Paris, France (M.V., P.B., S.T., Z.M.); 3 Department of Vascular Surgery (F.L.), University Hospital of Nice, and Université Côte d’Azur, France.; 4 Clinical Chemistry Laboratory (J.R.), University Hospital of Nice, and Université Côte d’Azur, France.

**Keywords:** angiotensin II, autophagy, clones, endoplasmic reticulum, inflammation, mice, smooth muscle cells

## Abstract

**Objective—:**

Recent studies suggested the occurrence of phenotypic switching of vascular smooth muscle cells (VSMCs) during the development of aortic aneurysm (AA). However, lineage-tracing studies are still lacking, and the behavior of VSMCs during the formation of dissecting AA is poorly understood.

**Approach and Results—:**

We used multicolor lineage tracing of VSMCs to track their fate after injury in murine models of Ang II (angiotensin II)–induced dissecting AA. We also addressed the direct impact of autophagy on the response of VSMCs to AA dissection. Finally, we studied the relevance of these processes to human AAs. Here, we show that a subset of medial VSMCs undergoes clonal expansion and that VSMC outgrowths are observed in the adventitia and borders of the false channel during Ang II–induced development of dissecting AA. The clonally expanded VSMCs undergo phenotypic switching with downregulation of VSMC differentiation markers and upregulation of phagocytic markers, indicative of functional changes. In particular, autophagy and endoplasmic reticulum stress responses are activated in the injured VSMCs. Loss of autophagy in VSMCs through deletion of autophagy protein 5 gene (*Atg5*) increases the susceptibility of VSMCs to death, enhances endoplasmic reticulum stress activation, and promotes IRE (inositol-requiring enzyme) 1α-dependent VSMC inflammation. These alterations culminate in increased severity of aortic disease and higher incidence of fatal AA dissection in mice with VSMC-restricted deletion of *Atg5*. We also report increased expression of autophagy and endoplasmic reticulum stress markers in VSMCs of human dissecting AAs.

**Conclusions—:**

VSMCs undergo clonal expansion and phenotypic switching in Ang II–induced dissecting AAs in mice. We also identify a critical role for autophagy in regulating VSMC death and endoplasmic reticulum stress–dependent inflammation with important consequences for aortic wall homeostasis and repair.

## Abstract 2

Supplemental Digital Content is available in the text.

**Highlights**

- Vascular smooth muscle cells (VSMCs) of the aortic media undergo clonal expansion in mouse models of dissecting aortic aneurysms.
- The clonally expanded VSMCs undergo phenotypic switching towards phagocytic-like phenotypes.
- Autophagy and endoplasmic reticulum stress responses are activated in some VSMCs. Autophagy in VSMCs control IRE (inositol-requiring enzyme)1α-dependent VSMC inflammation.
- We identify a critical role for autophagy in preserving vessel integrity and reducing the occurrence and severity of aortic dissection, possibly through limitation of VSMC death and endoplasmic reticulum stress–dependent inflammation.
- Our results suggest that promotion of proliferation and autophagy in VSMCs while inhibiting IRE1α-dependent inflammation may promote aortic wall repair and limit the development of dissecting aortic aneurysm.

The response of vascular smooth muscle cells (VSMCs) to injury is a major determinant of the development and progression of vascular diseases, including atherosclerosis, restenosis, and aneurysm.^1–3^ In response to injury and inflammation, VSMCs undergo phenotypic switching from a quiescent contractile phenotype to a proliferative, and migratory synthetic phenotype and can acquire molecular and cellular features of mesenchymal stem cells and macrophages.^4,5^ VSMC plasticity is well-documented during atherosclerosis and neointima formation and has been confirmed using lineage-tracing experiments.^6–9^ More recently, using multicolor lineage labeling, we demonstrated that VSMC accumulation in atherosclerotic plaques and injury-induced neointimal lesions results from extensive proliferation of a small subset of differentiated but highly plastic medial VSMCs, a variable proportion of which undergo phenotypic switching to phagocyte-like cells.^10^ VSMCs also play important roles in the pathophysiology of aortic aneurysm (AA), and recent studies suggested a role for some aspects of VSMC phenotypic switching in AA.^11,12^ However, the plasticity of VSMCs during AA formation has not been assessed.

**See accompanying editorial on page 982**

Here, we used multicolor lineage labeling of VSMCs to characterize the behavior of VSMCs during the development and progression of Ang II (angiotensin II)–induced dissecting AA. We report the occurrence of clonal expansion of a subset of VSMCs in the media, which can outgrow into the adventitia (including the false-channel’s borders) of the dissecting AA. The expanded VSMCs undergo phenotypic switching to phagocyte-like cells and can upregulate autophagy and endoplasmic reticulum (ER) stress markers. Importantly, loss of autophagy in VSMCs promotes VSMC death and ER stress–dependent VSMC inflammation and aggravates the aortic disease.

## Methods

### Data Disclosure Statement

The data that support the findings of this study are available from the corresponding authors on reasonable request.

### Animals

All experiments were performed according to the Home Office, UK regulations and approved by the local ethics committee. For lineage tracing, *Myh11-CreERt2/Rosa26-Confetti* males were subjected to 10 intraperitoneal injections of 1 mg tamoxifen over 2 weeks followed by at least 1-week washout. *Tagln*^*Cre+*^ mice (Jax n°004746) and *Atg5*^*flox/flox*^ mice (kindly provided by Noburu Mizushima, University of Tokyo^13^) were bred in house. *Tagln*^*Cre+*^/*Atg5*^*flox/flox*^ animals were used to assess the role of autophagy in VSMC. Infusion of 1µg/(min·kg) Ang II, with or without treatment with 10 mg/kg anti-TGF (transforming growth factor) β (clone 1.D.11, BioXCell) was used to induce dissecting AA. Animals were analyzed as described in the online-only Data Supplement.

### Statistical Analysis

Values are shown as average±SEM. Differences between groups were evaluated using Mann-Whitney test (2 groups), Kruskal-Wallis test followed by uncorrected Dunn test (> 2 groups), 2-way ANOVA (cell proliferation/survival), or χ^2^ test (distribution between 2 groups), as indicated in figure legends. Results were considered statistically significant at *P*<0.05.

## Results

### Characterization of VSMCs During Aortic Dissection Induced by Ang II

**Figure 1.**
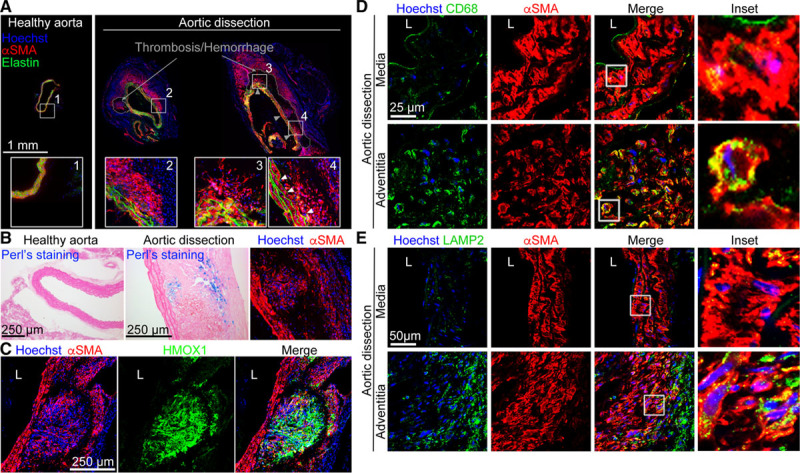
αSMA (α smooth muscle actin)^+^ cells accumulate outside of the media and express phagocytic makers after aortic dissection. **A**–**C**, Representative images of anti-αSMA staining on cross-sections of abdominal aortas from mice treated with Ang II and anti-TGF (transforming growth factor) β for 10 d showing αSMA^+^ cell outgrowths from the medial layer (**A**, **right**) as compared to sections from control mice (**A**, **left**). On consecutive sections, Perls (**B**) and anti-HMOX (heme oxygenase) 1 (**C**) staining show that αSMA^+^ cells in an iron-rich environment (Perls^+^) express the heme catabolic enzyme (HMOX1). **D**–**F**, Representative images of cryosections costained for αSMA and CD68 (**D**) or LAMP2 (lysosomal-associated membrane protein 2; **E**) showing that CD68 and LAMP2 colocalize with αSMA^+^ cells in the adventitial layers of aortic dissection. Hoechst (blue) represents nuclear staining. Images are representative of immunostainings done on 5 mice with aortic dissection.

**Figure 2.**
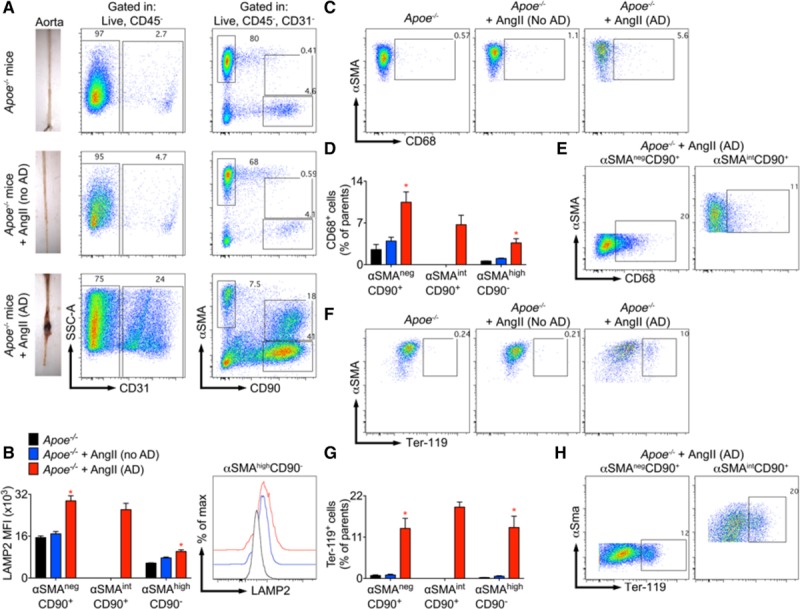
Vascular smooth muscle cells (VSMCs), fibroblasts, and myofibroblasts express phagocytic markers after aortic dissection (AD). Flow cytometric analysis of stromal cells (CD45^−^) in the aorta of 5-mo-old male *Apoe*^−/−^ mice infused with Ang II (angiotensin II) for 21 d (n=13). Littermate control *Apoe*^−/−^ mice were left untreated (**top**, n=4). **A**, **Left**, Representative images of aortic samples from untreated or Ang II–infused *Apoe*^−/−^ mice that either did not (no AD) or did develop an AD. Enzymatically digested aortas were analysed by flow cytometry. **Middle/right**, Representative dot plots showing the gating strategy used to analyze αSMA (α smooth muscle actin)^high^CD90^−^ cells (VSMCs), αSMA^-^CD90^+^ (fibroblast phenotype) and αSMA^low^CD90^+^ (myofibroblasts). Aortic dissection induced by Ang II (**lower**, n=4 mice) dramatically reduced the percentage of αSMA^high^CD90^-^ cells (VSMCs) within CD45^−^ cells but increased the percentages of αSMA^-^CD90^+^ (fibroblast phenotype) and αSMA^low^CD90^+^ (myofibroblasts). **B**, Quantification of LAMP2 (lysosomal-associated membrane protein 2) expression by αSMA^high^CD90^−^ cells (VSMCs), αSMA^−^CD90^+^ (fibroblast phenotype) and αSMA^low^CD90^+^ (myofibroblasts) and flow chart showing the expression of LAMP2 by αSMA^high^CD90^-^ cells (VSMCs). *P<0.05 Ang II (AD) vs Ang II (no AD) and untreated *Apoe*^−/−^ mice, Kruskal-Wallis test followed by Uncorrected Dunn test. **C**, Representative dot plots showing the percentage of αSMA^high^CD90^-^ cells (VSMCs) expressing CD68 in untreated Apoe^−/−^ mice (**left**) and Ang II treated *Apoe*^−/−^ mice without (**middle**) or with (**right**) AD. **D**, Quantification of CD68 expression by aortic αSMA^high^CD90^-^ cells (VSMCs), αSMA^-^CD90^+^ (fibroblast phenotype), and αSMA^low^CD90^+^ (myofibroblasts). *P<0.05 Ang II (AD) vs Ang II (no AD) and untreated *Apoe*^−/−^ mice, Kruskal-Wallis test followed by Uncorrected Dunn test. **E**, Representative dot plots showing the percentage of αSMA^-^CD90^+^ (fibroblast phenotype) and αSMA^low^CD90^+^ (myofibroblasts) expressing CD68 in dissected aortas. **F**, Representative dot plots showing the percentage of Ter-119 positive αSMA^high^CD90^-^ cells (VSMCs). **G**, Quantification of Ter-119 positive αSMA^high^CD90^-^ cells (VSMCs), αSMA^-^CD90^+^ (fibroblast phenotype), and αSMA^low^CD90^+^ (myofibroblasts). *P<0.05 Ang II (AD) vs Ang II (no AD) and untreated *Apoe*^−/−^ mice, Kruskal-Wallis test followed by Uncorrected Dunn test. **H**, Representative dot plots showing the percentage of Ter-119 positive αSMA^neg^CD90^+^ (fibroblasts) and αSMA^int^CD90^+^ (myofibroblasts) in aortas with AD.

VSMCs downregulate contractile gene expression during AA formation.^11,12^ However, the plasticity of VSMCs during AA formation has not been fully characterized. To this end, we used a prototypical model of dissecting AA induced by Ang II, with or without TGFβ inhibition.^14,15^ We first stained for αSMA (α smooth muscle actin) on cross-sections of mice with aortic dissections. We observed accumulation of αSMA^+^ cells in the false channel in 5 out of 5 animals displaying aortic dissection in this experiment. These αSMA^+^ cells seemed to expand from the media (Figure 1A) and accumulated in hemorrhagic/thrombotic areas in contact with iron (Perls staining, Figure 1B) and red blood cells, which may explain their acquisition of HMOX (heme oxygenase) 1 expression (Figure 1C). αSMA^+^ cells detected in the thrombotic/hemorrhagic region also showed increased expression of the phagocytic marker CD68 (Figure 1D) and the lysosomal marker LAMP2 (lysosomal-associated membrane protein 2; Figure 1E), suggesting that some VSMCs switched towards phagocyte-like cells. We further confirmed our results using flow cytometry analysis of aortic cells isolated from *Apoe*^−/−^ mice infused with Ang II for 21 days (Figure 2). The proportion of VSMCs (αSMA^high^CD90^-^) with high expression of αSMA markedly decreased in dissected aortas compared with controls, whereas a substantial proportion of αSMA^int^CD90^high^ (myofibroblasts) and αSMA^low^CD90^high^ (fibroblasts; Figure 2A) was observed in diseased aortas. VSMCs, myofibroblasts, and fibroblasts acquired phagocytic markers LAMP2 (Figure 2B) and CD68 (Figure 2C and 2D) proportionally to the severity of aortic disease, and cells from dissecting aneurysms were positive for Ter-119, suggesting an association with red blood cells (Figure 2F and 2G). These results suggest that a substantial proportion of VSMCs, myofibroblasts, and fibroblasts adopt a phagocyte-like phenotype in dissecting AA.

### VSMC Clonal Expansion and Phenotypic Switching in Dissecting AA

**Figure 3.**
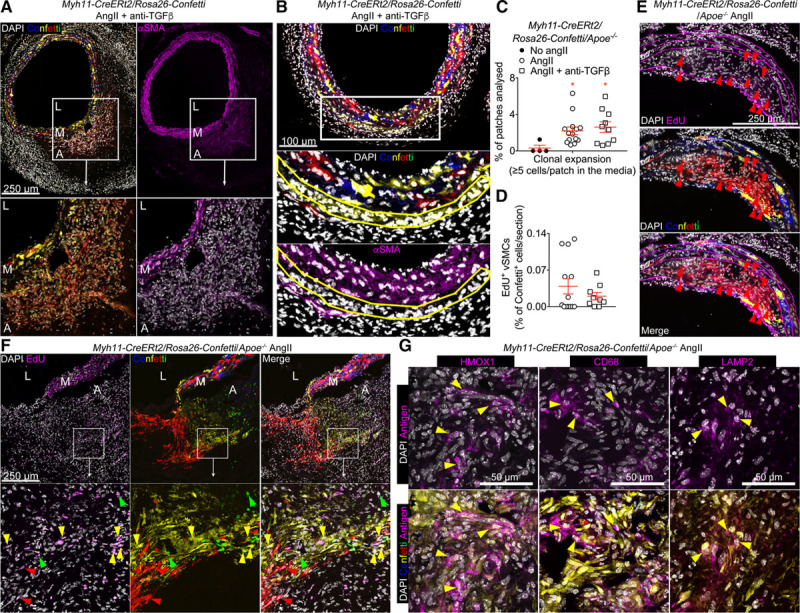
Vascular smooth muscle cells (VSMCs) undergo clonal proliferation and phenotypic switching on Ang II (angiotensin II) treatment and aortic dissection. **A** and **B**, Representative confocal images of abdominal aortic cross-sections from *Myh11-CreERt2/Rosa26-Confetti* animals treated with Ang II and anti-TGF (transforming growth factor) β. Signals for confetti colors (blue=CFP [cyan fluorescent protein], yellow=YFP [yellow fluorescent protein], red=RFP [red fluorescent protein], green=GFP [green fluorescent protein]), immunostaining for anti-αSMA (α smooth muscle actin; magenta), and DAPI (4’,6-diamidino-2-phenylindole; white) are shown as indicated. **A**, αSMA^+^ cells observed in the false channel of Confetti animals that developed an aortic dissection express the genetic Confetti lineage label, demonstrating that they are derived from Myh11-expressing VSMCs. Notably, the nonrandom color distribution of VSMC-derived cells in the adventitia indicates clonal expansion of a small number of cells. **B**, Monochromatic patches of VSMC-derived cells in the media express lower levels of αSMA compared with nonexpanding medial VSMCs. **C**, Quantification of clonal expansion in the media, represented as the fraction of monochromatic patches with ≥5 cells per patch, in the aorta of *Myh11-CreERt2/Rosa26-Confetti/Apoe*^−/−^ mice treated with Ang II alone or Ang II+anti-TGFβ, compared with healthy controls (no Ang II). Blocking TGFβ activity did not alter clonal VSMC expansion. Sixty-seven to 318 VSMC patches were analyzed per mouse. No Ang II: n=4, Ang II: n=14; Ang II+anti-TGFβ: n=10. *P<0.05 for No Ang II vs Ang II and No Ang II vs Ang II+anti-TGFβ. Data were analyzed using Kruskal-Wallis test followed by Uncorrected Dunn test. **D**, Quantification of EdU (5-ethynyl-2’-deoxyuridine) incorporation in VSMC-derived, Confetti-positive cells in *Myh11-CreERt2/Rosa26-Confetti/Apoe*^−/−^ mice infused with Ang II±anti-TGFβ and injected with EdU from day 14 to day 21 of Ang II infusion. Ang II: n=13; Ang II+anti-TGFβ: n=9. **E** and **F**, Representative confocal images of EdU staining in sections from Ang II–treated *Myh11-CreERt2/Rosa26-Confetti/Apoe*^−/−^ mice. Clonal expansion of VSMCs in the media (**E**) and adventitial outgrowth (**F**) is associated with DNA synthesis (proliferation) after Ang II infusion. Arrowheads (colored according to Confetti color) indicate EdU^+^ VSMCs. A low magnification image of **F** and **G** is available as Figure IV in the online-only Data Supplement**. G**, Representative images showing expression of HMOX (heme oxygenase) 1, CD68 and LAMP2 (lysosomal-associated membrane protein 2) in sections of a dissected aorta from *Myh11-CreERt2/Rosa26-Confetti/Apoe*^−/−^ mice infused with Ang II. Images show adventitial regions containing VSMC-derived cells and yellow arrowheads indicate Confetti-positive VSMC-derived cells expressing HMOX1, CD68, or LAMP2. A indicates adventitia; L, lumen; and M, media.

To test whether the αSMA^+^ cells that have accumulated in the adventitia have originated from preexisting VSMCs, we used multicolor lineage tracing in *Myh11-CreERt2/Rosa26-Confetti* mice to track VSMCs and their progeny.^10^ VSMCs were labeled by tamoxifen injections before the induction of AA by Ang II infusion and TGFβ inhibition (Figure 3). Stochastic labeling of VSMCs using this method results in a mosaic pattern in the noninjured aortic media.^10^ We found that αSMA^+^ cells that accumulated in the adventitia and the false channel in mice with aortic dissection were also positive for Confetti fluorescent reporters (5 out of 6 mice) indicating that they were VSMC-derived cells coming from the media (Figure 3A). Interestingly, in contrast to the stochastic mosaic labeling of the normal aortic media, VSMC-derived cells in the adventitia displayed a nonrandom color distribution (Figure 3A). We observed large regions containing lineage-labeled cells of a single color or intermixed single colors in all (5/5) animals with VSMC-derived Confetti^+^ cells outside the medial layer, suggesting that these cell outgrowths are derived from clonal expansion of a small number of cells (Figure 3A). We also found monochromatic patches of VSMCs in the medial layer of 5 out of the 6 animals analyzed (>5 cells per patch, Figure 3B and Figure I in the online-only Data Supplement), suggesting that proliferation is activated in a subset of medial VSMCs. The clonally expanded VSMCs observed in the adventitial outgrowths and in medial monochromatic patches significantly downregulated their αSMA expression (Figure 3A and 3B and Figure II in the online-only Data Supplement), further supporting that these cells undergo phenotypic switching. To examine whether the accumulation of VSMC-derived cells in the dissected area was a result of TGFβ inhibition, we lineage-traced VSMCs in *Apoe*^−/−^ animals that develop dissecting AA after Ang II treatment in the absence of TGFβ inhibition. The occurrence and extent of monochromatic patches were not affected by TGFβ neutralization in Ang II–treated animals (8/15 *Apoe*^−/−^ without anti-TGFβ and 6/10 *Apoe*^−/−^ with anti-TGFβ) (Figure 3C). Confetti-positive cells were observed in the thrombotic/hemorrhagic area of 3 out of 6 *Apoe*^−/−^ animals displaying limited aortic dissection (Figure III in the online-only Data Supplement). Analysis of EdU (5-ethynyl-2’-deoxyuridine) incorporation (Figure 3D) confirmed that VSMCs were proliferating both in the media (Figure 3E) and in the adventitial outgrowth areas of dissected aortas (Figure 3F and Figure IV in the online-only Data Supplement). Confetti^+^ cells in the media expressed almost no phagocytic markers, but the Confetti^+^ cells that have expanded into the adventitia started to express HMOX1, CD68, and LAMP2 (Figure 3G). CD90 expression was undetectable in Confetti^+^ cells, except for a few cells with very low expression (data not shown). Our data indicate that clonal proliferation and phenotypic switching of medial VSMCs are important features of Ang II–induced aortic dissection.

### *Atg5* Deficiency in VSMCs Promotes the Development of Severe Aortic Dissection

**Figure 4.**
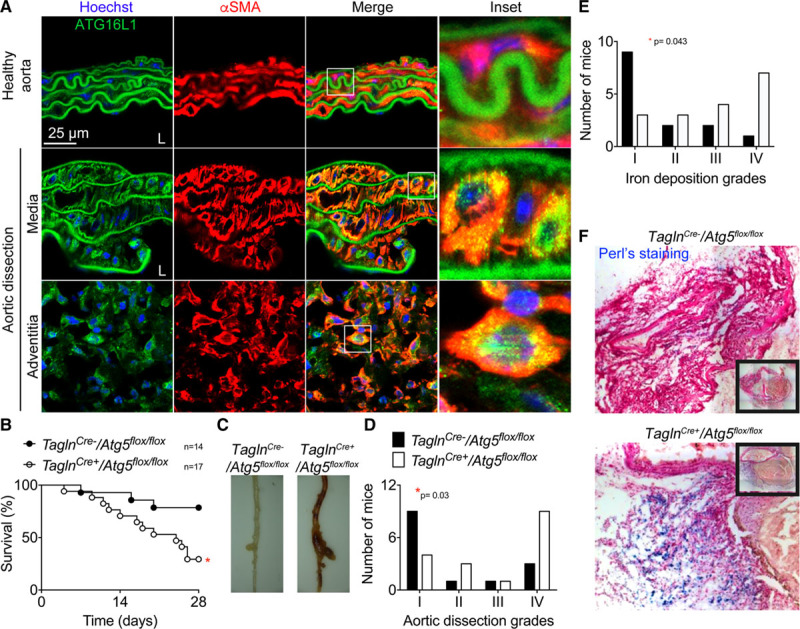
ATG5 (autophagy protein 5) deletion in vascular smooth muscle cell (VSMCs; *Talgn*^*Cre+*^/*Atg5*^*flox/flox*^ mice) increases susceptibility to aortic rupture induced by Ang II (angiotensin II) and anti-TGF (transforming growth factor) β infusion. **A**, Representative images of anti-αSMA (α smooth muscle actin) and anti-ATG16L1 (autophagy protein 16 like 1) staining of abdominal aortic cross-sections from untreated mice (healthy aorta, n=3) and mice infused with Ang II and anti-TGFβ that developed a dissection (n=5). αSMA^+^ cells (in the media and in the adventitial outgrowth) express elevated levels of ATG16L1 compared with healthy controls, and the staining shows a punctuate pattern, suggesting that autophagosomes are forming. **B**–**F**, *Tagln*^*Cre*-^ (n=14, black) and *Tagln*^*Cre+*^ (n=17, white) *Atg5*^*flox/flox*^ male littermate mice were infused with Ang II+anti-TGFβ. **B**, Survival curves. *P<0.05 *Tagln*^*Cre*-^ vs *Tagln*^*Cre+*^, Log-rank (Mantel-Cox) test. **C**, Representative images of thoraco-abdominal aorta. **D**, Severity of aortic dissections assessed macroscopically (I-normal appearance; II-thickening of the aortic wall; III- dissection; IV-fatal aortic rupture). *P<0.05 *Tagln*^*Cre*-^ vs *Tagln*^*Cre+*^, χ^2^ test. **E** and **F**, Quantification (**E**) and representative pictures (**F**) of iron deposition (blue Perls staining) in the aortic wall (I-no iron deposition; II-mild iron deposition; III-substantial iron accumulation in some cells; IV-high accumulation of iron in numerous cells). *P<0.05 *Tagln*^*Cre*-^ vs *Tagln*^*Cre+*^, χ^2^ test.

The lysosomal pathway, and particularly LAMP2, controls autophagosome maturation.^16^ Moreover, autophagy plays critical roles in VSMC biology^17^ and has recently been linked with VSMC phenotypic switching.^18^ Defective autophagy in VSMC is associated with accelerated VSMC senescence, neointima formation, and atherogenesis,^17,19^ but its role in the pathophysiology of dissecting AA is still uncertain.^20^ Studying aortic cross-sections, we found increased expression of ATG16L1 (autophagy-related protein 16 like 1) in medial and adventitial αSMA^+^ cells of dissecting AA (5 out of 5) compared with very limited staining in VSMCs of healthy aortas (Figure 4A), suggesting a potential role of autophagy in this disease condition. We confirmed that ATG16L1 is expressed in VSMC-derived cells, using the Confetti lineage tracing animals (Figure VA in the online-only Data Supplement). ATG5 (autophagy protein 5) is essential for all types of autophagy, and we found that ATG5 was also expressed in VSMC-derived Confetti^+^ cells (Figure VB and VC in the online-only Data Supplement). Furthermore, *Atg5* gene expression was upregulated in primary VSMCs at passage 4 in culture compared with ex vivo primary VSMCs (Figure VD in the online-only Data Supplement), further supporting a potential role of autophagy in phenotypically switched VSMCs. Using *Tagln*^*Cre+*^/*Atg5*^*flox/flox*^ mice (Figure VIA in the online-only Data Supplement), we investigated the impact of defective autophagy in VSMCs on the incidence of aortic dissection in mice. There was no difference in the blood pressure response to Ang II between *Tagln*^*Cre+*^/*Atg5*^*flox/flox*^ and *Tagln*^*Cre*-^/*Atg5*^*flox/flox*^ mice (Figure VIB in the online-only Data Supplement). Over 28 days, mice with *Atg5* deficiency in VSMCs showed reduced survival compared with their wild-type littermates (Figure 4B). Nine out of 17 *Tagln*^*Cre+*^/*Atg5*^*flox/flox*^ mice died from aortic rupture compared with 2 out of 14 *Tagln*^*Cre*-^/*Atg5*^*flox/flox*^ mice (Figure 4C and 4D). Moreover, 3 out of 17 *Tagln*^*Cre+*^/*Atg5*^*flox/flox*^ mice died without evidence of aortic rupture at autopsy but instead presented with hemorrhage in the peritoneum, spleen, and intestine, suggesting vascular impairment in those organs. Analysis of aortic tissue samples showed that vascular injury induced by Ang II+anti-TGFβ was significantly more severe in mice with VSMC-restricted *Atg5* deletion (Figure 4D), with higher levels of iron deposition (blue Perls staining) in the media and adventitia (Figure 4Eand 4F) as compared to their littermate controls. Thus, defective autophagy in VSMCs increases the incidence and severity of aortic dissection.

### *Atg5* Deficiency in VSMCs Impedes Autophagosome Formation and Enhances Cell Death

**Figure 5.**
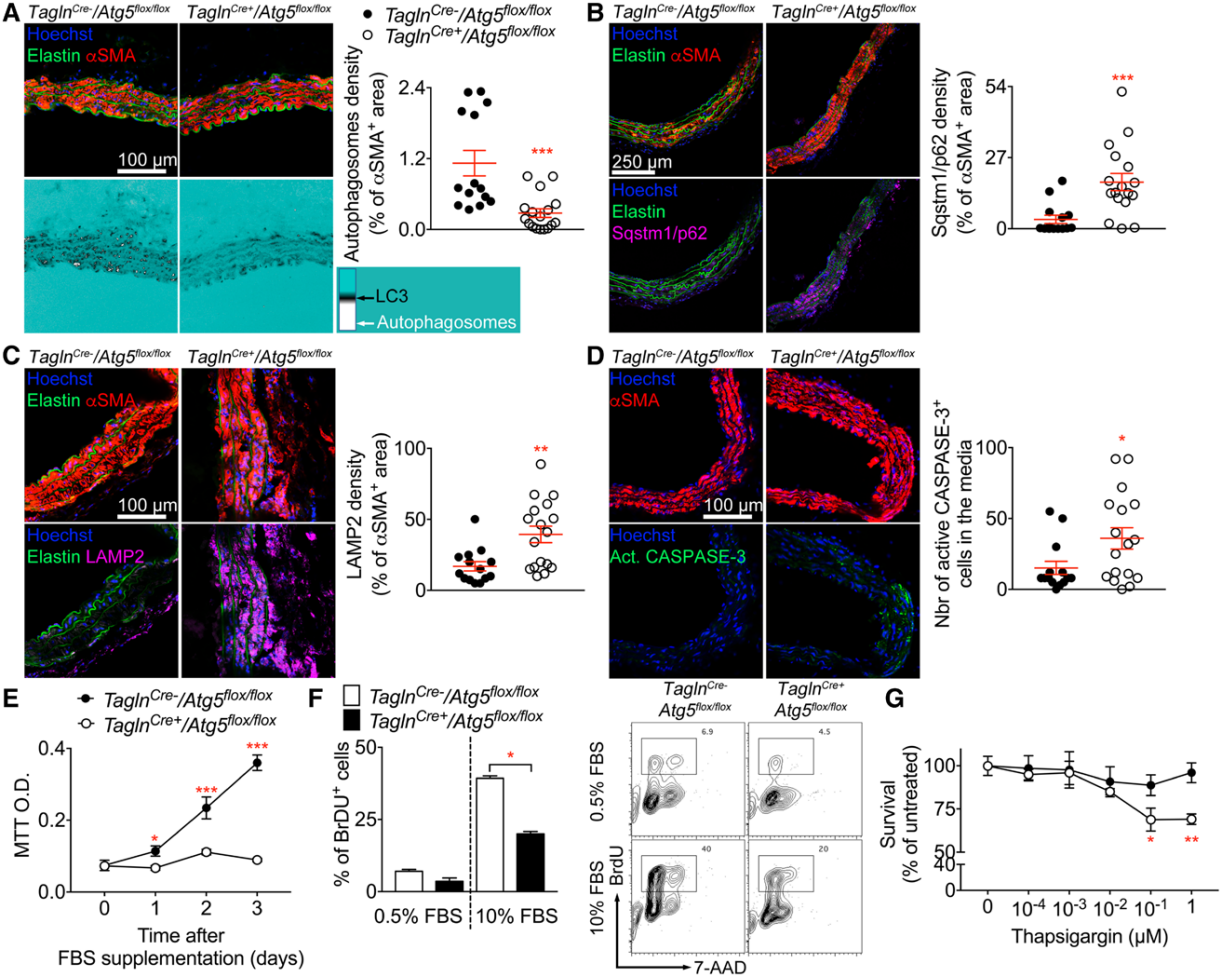
Impaired autophagy in vascular smooth muscle cells (VSMCs) alters autophagosome formation and enhances cell death. **A**–**D**, Aortic cross-section from *Tagln*^*Cre*-^ (n=14, black) and *Tagln*^*Cre+*^ (n=17, white) *Atg5*^*flox/flox*^ mice infused with Ang II (angiotensin II)+anti-TGFβ for 28 d were analyzed by confocal microscopy. **A**, Representative images showing αSMA (α smooth muscle actin) staining (**top**) and LC3 (microtubule-associated protein 1 light chain 3) signal (**lower**). The quantification of autophagosome formation in VSMCs was done using a filter showing background in Cyan, low LC3 staining appears in black, and LC3 bright spots in white (autophagosomes). ***P<0.001 *Tagln*^*Cre*-^ vs *Tagln*^*Cre+*^, Mann-Whitney test. **B**, Representative images showing αSMA (**top**) and p62 staining (**lower**). Quantification of p62 signal in αSMA-positive VSMCs is shown to the **right**. ***P<0.001 *Tagln*^*Cre*-^ vs *Tagln*^*Cre+*^, Mann-Whitney test. **C**, Representative images showing LAMP2 (lysosomal-associated membrane protein 2) and αSMA staining and quantification of lysosome accumulation in VSMCs. **P<0.01 *Tagln*^*Cre*-^ vs *Tagln*^*Cre+*^, Mann-Whitney test. **D**, Representative images showing αSMA and active CASPASE-3 staining. Quantification of the number of active CASPASE-3–positive apoptotic cells in the media is shown on the **right**. *P<0.05 *Tagln*^*Cre*-^ vs *Tagln*^*Cre+*^, Mann-Whitney test. **E** and **F**, Primary VSMCs were derived from the aorta of *Tagln*^*Cre*-^ and *Tagln*^*Cre+*^ *Atg5*^*flox/flox*^ mice and cultured for 4 to 7 passages before analysis. Mean±SEM of technical quadruplicates are shown. **E**, Serum-starved VSMCs were stimulated with serum and cell density analyzed by MTT (3-(4,5-dimethylthiazol-2-yl)-2,5-diphenyltetrazolium bromide) assay. *P<0.05, ***P<0.001 *Tagln*^*Cre*-^ vs *Tagln*^*Cre+*^ at each time point, 2-way ANOVA followed by uncorrected Fisher test. **F**, BrdU (5-bromo-2’-deoxyuridine) incorporation by serum starved VSMCs and cells supplemented with FBS for 48 h. *P<0.05 *Tagln*^*Cre*-^ vs *Tagln*^*Cre+*^ with 10% FBS, Mann-Whitney test. **G**, VSMCs were incubated with increasing doses of thapsigargin for 16 h. Cell density was analyzed using MTT assay and normalized to untreated *Tagln*^*Cre*-^ cells. **P<0.01, ***P<0.001 *Tagln*^*Cre*-^ vs *Tagln*^*Cre+*^ at each time point, 2-way ANOVA followed by uncorrected Fisher test. AAD indicates aminoactinomycin D; Act, active; and O.D., optical density.

To confirm that *Atg5* deficiency inhibits autophagy in VSMCs, we analyzed the expression of LC3 (microtubule-associated protein 1 light chain 3) in αSMA^+^ cells after the induction of AA. Punctate LC3 staining, associated with autophagosome formation, was significantly reduced in *Tagln*^*Cre+*^/*Atg5*^*flox/flox*^ mice compared with *Tagln*^*Cre*-^/*Atg5*^*flox/flox*^ control animals (Figure 5A). Conversely, *Atg5* deficiency in VSMCs led to a significant accumulation of the autophagosome cargo protein SQSTM1 (sequestosome 1)/p62 (Figure 5B) as well as LAMP2 (Figure 5C). Loss of *Atg5* was associated with an increase of apoptotic VSMCs in the media, as shown by active CASPASE-3 staining (Figure 5D), suggesting that autophagy promotes cell survival. This was confirmed using in vitro experiments, which revealed a substantial reduction of VSMC survival (Figure 5E) and proliferation (Figure 5F) in response to serum, and an increased susceptibility to ER stress–induced cell death (Figure 5G) in the absence of *Atg5*.

### *Atg5* Deficiency in VSMCs Promotes an ER Stress Response and Inositol-Requiring Enzyme 1α–Dependent Inflammation

**Figure 6.**
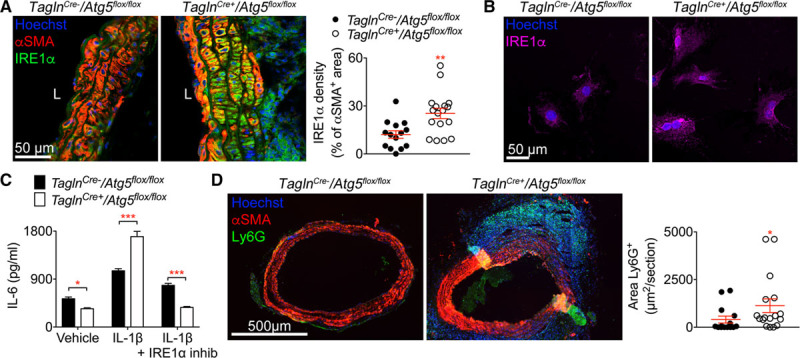
ATG5 (autophagy protein 5) deficiency in vascular smooth muscle cells (VSMCs) promotes inflammation via the endoplasmic reticulum stress sensor IRE (inositol-requiring enzyme) 1α. **A**, Representative images and quantification of IRE1α immunofluorescence signals in αSMA (α smooth muscle actin)^+^ cells on aortic cross-sections from *Tagln*^*Cre*-^ (n=14, black) and *Tagln*^*Cre+*^ (n=17, white) *Atg5*^*flox/flox*^ mice infused with Ang II (angiotensin II) and anti-TGF (transforming growth factor) β for 28 d. **P<0.01 *Tagln*^*Cre*-^ vs *Tagln*^*Cre+*^, Mann-Whitney test. **B**, Representative images of IRE1α immunostaining of primary VSMCs derived from the aorta of *Tagln*^*Cre*-^ and *Tagln*^*Cre+*^ *Atg5*^*flox/flox*^ mice cultured in vitro. **C**, IL (interleukin)-6 secretion by primary VSMCs derived from the aorta of *Tagln*^*Cre*-^ and *Tagln*^*Cre+*^ *Atg5*^*flox/flox*^ mice stimulated for 16 h with IL1β (100 pg/mL) in the presence or absence of the IRE1α kinase inhibitor (Apy29, 20 µM) in vitro. Mean±SEM of technical quadruplicates are shown. *P<0.05, ***P<0.001 *Tagln*^*Cre*-^ vs *Tagln*^*Cre+*^, 2-way ANOVA followed by uncorrected Fisher test. **D**, Representative images and quantification of Ly6G immunostaining on aortic cross-sections from *Tagln*^*Cre*-^ (n=14) and *Tagln*^*Cre+*^ (n=17) *Atg5*^*flox/flox*^ mice infused with Ang II+anti-TGFβ for 28 d. *P<0.05 *Tagln*^*Cre*-^ vs *Tagln*^*Cre+*^, Mann-Whitney test.

There is a close interplay between autophagy and the ER stress response, and recent studies indicate that autophagy may resolve ER stress responses through direct removal of IRE (inositol-requiring enzyme)1α.^21^ Consistent with the latter finding, we observed a substantial accumulation of the ER stress sensor IRE1α in *Atg5*-deficient VSMCs in vivo (Figure 6A). In vitro cultured *Atg5*-deficient VSMCs also showed substantial accumulation of IRE1α in the absence of any external stimulus (Figure 6B). VSMCs respond to IL (interleukin) 1 stimulation by abundant secretion of inflammatory cytokines^22^ and chemokines.^23^ Interestingly, IL1β-induced expression of *Il6* (Figure 6C), *Cxcl1* (C-X-C motif chemokine ligand 1), and *Ccl2* (C-C motif chemokine ligand 2; Figure VII in the online-only Data Supplement) was significantly higher in *Atg5*-deficient VSMCs compared with wild-type control cells and was abrogated by inhibition of IRE1α kinase activity. Consistent with the increased inflammatory response, aortic sections of *Tagln*^*Cre+*^/*Atg5*^*flox/flox*^ mice treated with Ang II+anti-TGFβ showed increased neutrophil accumulation compared with *Tagln*^*Cre*-^/*Atg5*^*flox/flox*^ control mice (Figure 6D). We also found a tendency (*P*=0.06) towards increased circulating levels of IL-6 in *Tagln*^*Cre+*^/*Atg5*^*flox/flox*^ compared with *Tagln*^*Cre*-^/*Atg5*^*flox/flox*^ mice; however, other tested circulating cytokines and chemokines were not different between the 2 groups (Figure VII in the online-only Data Supplement). These results indicate that autophagy-dependent regulation of ER stress modulates VSMC and local aortic inflammation.

### Autophagy and ER Stress Are Features of Human Aortic Dissection

**Figure 7.**
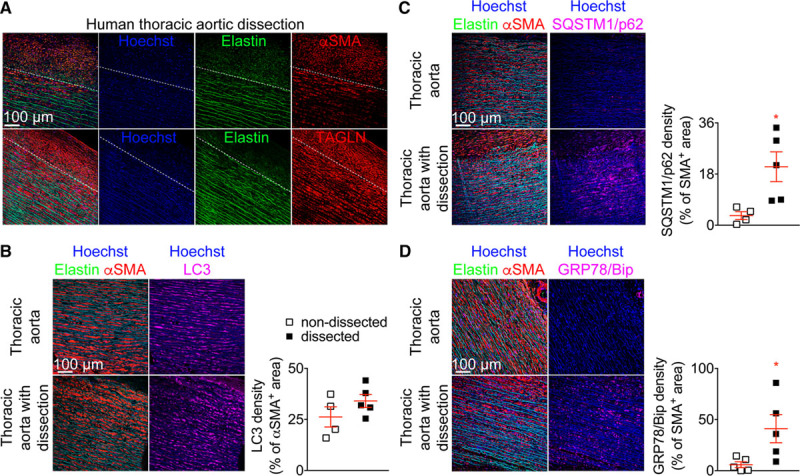
Human aortic dissections are associated with impaired autophagy and endoplasmic reticulum stress response in vascular smooth muscle cells. **A**–**D**, Human thoracic aortic samples from nondissected (n=4–5) and dissected (n=5) aortas were immunostained and analyzed by confocal microscopy. **A**, Representative images showing that αSMA (α smooth muscle actin)^+^ and TAGLN^+^ (transgelin) cells are detected outside the media (dotted line depicts the external elastic laminae) in the adventitial layer of dissected thoracic aorta (4 out of 5 samples) but not in nondissected samples (0 out of 5 samples). **B**–**D**, Representative images and quantification of the expression of LC3 (microtubule-associated protein 1 light chain 3; **B**), SQSTM1 (sequestosome 1)/p62 (**C**) and GRP78/BiP (glucose-regulated protein 78/binding immunoglobulin protein; **D**) in αSMA^+^ cells in the media of nondissected (n=4–5, white) and dissected aortic samples (n=5, black). *P<0.05 nondissected vs dissected, Mann-Whitney test.

To examine the relevance of our findings to human pathology, we analyzed sections of human aortas with or without dissection, collected from separate patients. We found that 4 out of 5 samples with aortic dissection contained αSMA^+^ and TAGLN^+^ (transgelin) cells (Figure 7A) in areas devoid of elastic lamellae outside the media, whereas such features could not be detected in nondissected normal aortas (n=5; Figure VIII in the online-only Data Supplement). The vast majority of adventitial αSMA^+^ cells did not express CD90 (Figure IX in the online-only Data Supplement) indicating that they were not of fibroblast origin and suggesting that they have most likely expanded from the aortic media. Although LC3 expression in VSMCs was similar between nondissected and dissected aortas (Figure 7B), the latter showed increased accumulation of the autophagosome cargo protein SQSTM1/p62 (Figure 7C) and increased expression of the ER stress marker GRP78/BiP (glucose-regulated protein 78/binding immunoglobulin protein; Figure 7D). Our data suggest that VSMCs of dissected AAs in humans may have deregulated autophagy resulting in ER stress activation.

## Discussion

Previous work on the role of VSMCs in AA has focused on the detrimental effects of VSMC death in promoting adverse arterial wall remodeling because of reported medial thinning, degeneration, and extensive apoptosis of VSMCs in very late stages of AA development.^24,25^ Notably, however, despite medial thinning and VSMC death, ascending thoracic AAs show an increase in overall medial area and have preserved VSMC density, suggesting a hyperplastic response.^26,27^ Animal models of AA also suggest that VSMCs display some aspects of phenotypic switching early during the development of both thoracic and abdominal AA.^11,12^ Here, we have tested the hypothesis that a hyperplastic VSMC response could compensate for increased VSMC death during AA development. Using lineage tracing of preexisting VSMCs in mice, we found that in response to Ang II infusion, a subset of VSMCs clonally expand in the media of the thoracic and abdominal aorta, and in the context of aortic dissection, expand through the aortic wall into the adventitia and the newly formed false channel. We propose that the resulting VSMC-derived cells might play a reparative role at several disease stages, from aneurysm development to aneurysm dissection. Interestingly, large foci of VSMCs also accumulate in areas of extensive elastin degradation corresponding to the external medial layers and adjacent adventitia of human thoracic AAs, suggesting similar pathophysiological mechanisms in human AAs.

Our lineage-tracing experiments demonstrate that a subset of preexisting VSMCs proliferate, downregulate contractile protein expression, and upregulate proteins associated with a phagocytic-like phenotype in AA. This resembles the VSMC behavior observed in other vascular disease models,^7–10^ suggesting that the extensive plasticity is an inherent physiologically relevant feature of VSMCs. Importantly, we found many examples of clonal proliferation resulting in monochromatic patches within the medial layer in animals showing no signs of aortic dissection. This observation suggests that activation of proliferation occurs in a larger proportion of VSMCs than what was estimated from the clonal VSMC contribution to neointima formation after vascular injury.^10^

The finding that VSMCs downregulate the contractile phenotype in AA is consistent with and further validates previous work.^11,12,28^ Two recent studies reported that interference with molecular pathways involved in VSMC phenotypic switching may have detrimental effects in AA. VSMC-restricted deletion of KLF4 (Kruppel-like factor 4), which has previously been identified as a regulator of several aspects of VSMC phenotypic switching in atherosclerosis,^8^ reduced aortic disease severity in mouse models of AA,^28^ although it did not abrogate the disease. A more recent study identified a role for HDAC9-MALAT1-BRG1 (histone deacetylase 9-metastasis-associated lung adenocarcinoma transcript 1-brahma-related gene 1) complex in the downregulation of the contractile VSMC phenotype in AAs driven by mutations of the TGFβ pathway; VSMC-restricted deletion of MALAT1 significantly preserved the contractile phenotype of VSMCs and reduced AA development in a mouse model of Marfan with *Fbn1* mutation.^12^ These studies are consistent with a detrimental role of the downregulation of the contractile phenotype of VSMCs in AAs. However, KLF4 and MALAT1 may impact other VSMC functions beyond, and may be independently, of their role in regulating the contractile phenotype of VSMCs.

Beyond the downregulation of differentiation and contractile markers of VSMCs, VSMC phenotypic switching induces a wide range of functions, which might have opposing functions on AA formation and progression. Here, we assessed the particular role of VSMC autophagy in AA and found that loss of *Atg5* in VSMC reduced autophagosome generation and resulted in increased disease progression and mortality in Ang II–treated animals with TGFβ inhibition. Previously, the role of VSMC autophagy in the development of AA was examined in *Atg7*^*flox/flox*^/*Tagln*^*Cre/+*^ mice.^20^ The authors concluded that mice with smooth muscle cell–specific *Atg7* deficiency do not develop dissecting abdominal AA in response to Ang II.^20^ Importantly, that study was conducted using Ang II infusion under normocholesterolemic conditions where mice are resistant to AA.^14,15^ Additional cues, such as the presence of hypercholesterolemia^14^ or the concomitant blockade of TGFβ signaling pathway,^15,29^ are required to promote the susceptibility of the aorta to aneurysm formation and dissection in response to Ang II infusion. In our present study, the use of a previously validated model of dissecting AA^15,29^ revealed a clear detrimental effect of defective autophagy in VSMCs on AA development. The incidence and severity of dissected AAs were significantly higher in mice with *Atg5* deletion in VSMCs. Of note, 18% of the mice (25% of the mice that died suddenly) showed evidence of extraaortic hemorrhage in the peritoneum, spleen, and intestine, suggesting that defective autophagy in VSMCs may be associated with widespread impairment of the vascular response to injury. Taken together, the data show that autophagy in VSMCs is critically required for the maintenance of vascular integrity during the development and progression of AAs. This vasculoprotective effect may be explained at least in part, by the role of autophagy in preserving VSMC survival in response to injury. Our data also identify a role of autophagy in the regulation of VSMC inflammation, potentially through the degradation of IRE1α.^21^ IRE1α has previously been involved in mediating inflammatory responses downstream of toll-like receptors^30,31^ and C-type lectin receptors,^32^ but its role in IL1R1 (interleukin 1 receptor 1) signaling pathways has not been addressed. We speculate that this interconnection between autophagy, ER stress responses, and inflammatory pathways is of major importance to the outcome of the reparative process after injury and merits further consideration. Finally, the direct impact of autophagy on the regulation of VSMC clonal proliferation and phenotypic switching will need to be addressed. It will also be interesting to address the direct impact of autophagy deletion on the response of VSMC to Ang II stimulation.

## Conclusions

We provide genetic evidence for the activation of VSMC proliferation, selective clonal expansion, and phenotypic switching towards phagocytic-like phenotypes in VSMCs during the development of dissecting AA. We identify a critical role for autophagy in the preservation of vessel integrity, possibly through limitation of VSMC death and ER stress–dependent inflammation. The results advance our understanding of the reparative mechanisms that operate during aneurysm development and progression, which could be exploited clinically. Future studies to identify the precise stimuli responsible for VSMC proliferation and accumulation in this context are important to reveal potential new therapeutic targets.

## Acknowledgments

We thank the Wellcome Trust–Medical Research Council, Institute of Metabolic Science, Metabolic Research Laboratories, Imaging core, Wellcome Trust Major Award (208363/Z/17/Z), and the Cambridge National Institute for Health Research Biomedical Research Centre Cell Phenotyping Hub.

## Sources of Funding

Z. Mallat is supported by the British Heart Foundation, United Kingdom. The project also received funding from INSERM, France. J. Chappell and A.L. Taylor are supported by British Heart Foundation (BHF) studentships (RE/13/6/30180, FS/14/59/31282). H.F. Jørgensen is supported by the BHF Cambridge Centre for Research Excellence (RE/13/6/30180) and the BHF Oxbridge Centre for Regenerative Medicine (RM/13/3/30158, RM/17/2/33380).

## Disclosures

None.

## Supplementary Material
