# Supplementary material for: Vascular Smooth Muscle Cell Plasticity and Autophagy in Dissecting Aortic Aneurysms
Source: Arterioscler Thromb Vasc Biol. 2019 Apr 4;39(6):1149–59. doi: 10.1161/ATVBAHA.118.311727 (PMC6544538; doi:10.1161/ATVBAHA.118.311727)
Supplement: Supplementary file 1 [file atv-39-1149-s001.pdf]

SUPPLEMENT MATERIAL

Vascular Smooth Muscle Cell Plasticity in Dissecting Aortic Aneurysms

Clement et al.

SUPPLEMENTAL METHODS

Animals

All the experiments were approved by the local ethics committee and done under Home Office, UK licenses PA4BDF775 and 70/7555.

In order to lineage label VSMCs, *Myh11-CreERt2/ROSA26-Confetti and Myh11-CreERt2/ROSA26-Confetti/Apoe$^{-/-}$* male mice (previously described in Chappell et al.[1]), received 10 injections of tamoxifen diluted in corn oil (1mg/mouse/injection, i.p.) over 2 weeks. Mice were left for a week to clear excess tamoxifen, before further treatment.

Myh11-CreERt2/ROSA26-Confetti mice were infused with AngII (1µg/min/Kg, Sigma) using osmotic pumps and anti-TGFβ (10mg/Kg, clone 1.D.11, BioXCell) was injected i.p. (3 times/week) and tissues were harvested at different time points between day 7 and day 28. Out of 22 animals treated with AngII, 4 died within one week and were excluded from the analysis. Of the remaining, 6 animals showed macroscopic evidence of aortic dissection and were analyzed for VSMC phenotype by confocal microscopy. Additionally, one of the animals which appeared to have a normal aorta was also analyzed by confocal microscopy.

Myh11-CreERt2/ROSA26-Confetti/Apoe$^{-/-}$ mice were infused with AngII (1µg/min/Kg) using osmotic pumps, and inhibition of TGFβ activity (10mg/Kg, clone 1.D.11, 3 times/week, i.p.) started 14 days after the beginning of AngII infusion. Mice were injected daily with EdU (1mg/mouse) i.p. for a week, starting on day 14. A total of 25 animals were treated with AngII in three independent experiments and 10 of these received anti-TGFβ injections (5 animals per experiment in two experiments). All animals were processed for analysis by confocal microscopy.

For flow cytometry experiments, 5-month-old male *Apoe$^{-/-}$* mice were subjected to AngII infusion (n=13), or left untreated (n=4), for 21 days later. 2 out of the 6 mice infused with AngII developed an aortic dissection. Aortic tissues were harvested,

dispersed enzymatically to a single cell suspension as described [2] and analyzed by flow cytometry.

Tagln^{Cre+} mice (Jax n°004746) and *Atg5*$^{flox/flox}$ mice (kindly provided by Noburu Mizushima, University of Tokyo [3]) were crossed and male littermates *Tagln*$^{Cre+}$ (n=14) and *Tagln*$^{Cre-}$ (n=17) on *Atg5*$^{flox/flox}$ background were infused with AngII (1µg/min/Kg, Sigma) and anti-TGFβ (10mg/Kg, clone 1.D.11, 3 times/week, i.p.). Data are from 2 independent experiments. Blood pressure measurements were obtained by the tail cuff method. Necropsies were performed to confirm vascular rupture of animals that died prior to day 28, and the aortas were harvested and fixed in 4% PFA. Surviving mice were culled at day 28, perfused with cold PBS and aortic samples were fixed in PFA 4% overnight at 4°C, and kept in PBS at 4°C for further investigation. Assessment of aortic dissection stage (I-normal appearance; II-thickening of the aortic wall; III-dissection; IV-fatal aortic rupture) was done blinded to genotype.

Tissue processing and quantification of medial patches in lineage-traced animals. Aortas were fixed in 4% PFA overnight at 4°C, embedded in OCT and cryosectioned (12 µm) as described in Chappell et al. [1]. Sections were stained for antigens of interest, EdU incorporation and the nuclei were counter stained with DAPI as described [1] using the primary antibodies listed below, except for αSMA stainings that were done using a biotinylated primary antibody (Abcam clone 1A4) and Alexa Fluor® 647 Streptavidin (Biolegend). Secondary antibodies were all Alexa Fluor® 647 conjugated to avoid spectral overlap with the Confetti reporter proteins. Sections were mounted in RapiClear and imaged using an Sp8 Leica confocal microscope as previously described [1], with a 2.5 µm distance between scans. The number of EdU+ cells per section was quantified in Imaris (Bitplane, Oxford Instruments) to ensure that EdU+ nuclei were scored correctly for Confetti signal. Quantification of Confetti patch size within the media was performed in imaged sections within the Imaris section viewer, with Z-stack thickness of 3.5 µm. Within each "ring" of the artery (delineated by the elastic lamella) each cell was scored for its Confetti color (or absence of) cell by cell and the frequency of occurrence of contiguous runs of one Confetti color calculated for each patch size.

Immunofluorescence. Aortic samples were cleaned of surrounding tissues, embedded in OCT and cryosectioned. Immunofluorescent stainings were performed as described in Clement et al. [2]. Sections were stained with mouse anti-mouse/human αSMA-Cy3™ (Sigma, clone: 1A4), mouse anti-mouse αSMA-biotin (Abcam, ab125057), rabbit anti-mouse HMOX1 (Abcam, clone: EP1391Y), rat anti-mouse CD68 (Biorad clone: FA-11), rat anti-mouse LAMP2 (SantaCruz biotech, clone: M3/84), rabbit anti-mouse ATG16L1 (Cell Signaling Technology®, clone: D6D5), rabbit anti-mouse ATG5 (LifeSpan BioSciences LS-B1843), rabbit IgG control (Abcam ab27478), rabbit anti-mouse/human LC3 (Cell Signaling Technology®, clone: D11), rabbit ant-mouse/human Sqstm1/p62 (Abcam, ab207305), rabbit anti-mouse active CASPASE3 (Cell Signaling Technology®, clone: D3D9), rabbit anti-mouse/human IRE1α (Cell Signaling Technology®, clone: 14C10), rat anti-mouse Ly6G (eBiosciences, clone: RB6-8C5), rabbit anti-human GRP78/Bip (Cell Signaling Technology®, clone: C50B12) and rabbit anti-human TAGLN (Abcam, ab14106). Primary antibodies were revealed using donkey anti-rabbit Alexa Fluor® 555 (Invitrogen), goat anti-rat Alexa Fluor® 488 (Invitrogen) or goat anti-rabbit Alexa Fluor® 647 (Invitrogen, and Abcam ab150079).

EdU incorporation was detected using the Click-iT® Plus EdU Alexa Fluor® 647 Imaging kit (Life Tehcnologies) according to the manufacturer's instructions.

Sections from human aortic samples were obtained by P. Bruneval, Paris, France. At the time of collection, those samples were considered waste necropsy or post-surgery material and did not require specific ethics approval. Human samples from non-dissected thoracic aortas (n=5) were obtained from men (age 44-68 years old) suffering from traumatic injury of the ascending aorta (n=1), valvular surgery (n=1) or from transplantation-recused hearts (n=3). Tissues from dissected thoracic aortas (n=5; 4 ascending, 1 descending) were obtained from men (age 53-75 years old) suffering from chronic (n=3) or acute dissection (n=2) associated with degenerative disease (no bicuspid valves). Deparaffinization and staining were performed as described in Clement et al. [2]. Imaging of epifluorescent and brightfield stainings was performed using a Leica DM6000B microscope, and images were analyzed using Adobe Photoshop CS5 and ImageJ (NIH). Immunofluorescent imaging by confocal

microscopy was done using a Carl Zeiss LSM 700 confocal microscope and Zen2009 software.

Perl's staining and assessment of iron accumulation. Sections were stained according to the manufacturer's instructions (Sigma, HT20-1KT). Iron staining was graded as follow: I-no iron deposition; II-mild iron deposition; III-high iron accumulation in some cells; IV-high accumulation of iron in numerous cells.

Flow cytometry. Aortic samples were digested and cell suspensions were stained following the protocol described in Clement et al. [2]. Extracellular antigens were stained using rat anti-mouse CD45-eVolve605^{TM} (eBiosciences, clone: 30F11) or anti-CD45-APC, (Biolegend, clone: 30F11), rat anti-mouse CD31-PE-Cy7 (eBiosciences, clone: 390), rat anti-mouse CD90.2-BV786^{TM} (BD Biosciences, clone: 53-2.1) and Zombie Yellow™ Fixable Viability Kit (Biolegend). Cells were then fixed/permeabilized using a transcription factor staining buffer set (eBioscience™ Foxp3) according to the manufacturer's instructions, and intracellular antigens were stained using mouse anti-mouse αSMA-Cy3™ (Sigma, clone: 1A4), rat anti-mouse CD68-BV605^{TM} (Biolegend, clone: FA-11), rat anti-mouse LAMP2-FITC (eBiosciences, clone: eBioABL-93), anti-Ter-119-FITC (eBiosciences, clone: Ter-119). Flow cytometric acquisition of the cell suspension was performed on a LSR II Fortessa (BD biosciences) equipped with 4 lasers (405, 488, 561 and 640 nm). Analysis was done using BD FACSDiva Software 6.0 and figure-displayed dot plots and histograms were obtained using FlowJo software (TreeStar).

Cell culture. VSMC primary cultures were obtained using aortas from female *Atg5*$^{flox/flox}$ *Tagln*$^{Cre-}$ and *Tagln*$^{Cre+}$ littermates. Briefly, aortas were cleaned from surrounding tissues, minced into small pieces, and subjected to enzymatic digestion (RPMI 1640, collagenase D [0.2 mg/ml, Roche], dispase [1 U/ml, Stemcell^{TM} Technologies] and elastase [1 mg/ml, Worthington biochemical Corporation]) for 45 min at 37°C. Cells were allowed to grow in complete medium (RPMI 1640 containing L-glutamine + 10 % [vol/vol] heat-inactivated FBS, 100 IU/ml penicillin, 100 µg/ml streptomycin) until passage 4 and were then aliquoted and frozen in liquid nitrogen. Serum induced proliferation of VSMC was done after overnight serum starvation (0.5% serum) and complete medium was added onto the cells. Cell density was

evaluated using MTT assays (CGD1-1KT, Sigma). For DNA synthesis analysis, cells were incubated with in the same conditions, and the medium (0.5% or 10% FBS) was supplemented with BrdU (1mM). Cells were harvested 48 hours after FBS supplementation and BrdU incorporation was analyzed by flow cytometry according the manufacturer’s instruction (BD Biosciences, Kit BrdU, cat number: 559619). IL-6 production after IL-1β stimulation was performed on cells plated at $1x10^5$ cells/well, left overnight to adhere in complete medium. Cells were washed and stimulated with fresh medium containing either 0.1% DMSO (vehicle), vehicle + IL-1β (100 pg/ml, Biolegend), or IL-1β + Apy29 (20 µM, Tocris). After 24 hours, supernatants were collected and IL-6 secretion was analyzed using CBA Mouse IL-6 Flex Set (BD Biosciences). Death induced by thapsigargin (Sigma) was measured on VSMCs plated at $1x10^5$ cells/well, left overnight to adhere in complete medium. Medium was replaced with fresh medium containing increasing concentration of thapsigargin (0-1µM). After 24 hours, cell density was assessed using MTT assay. Tests were performed in quadruplicate.

Quantitative real time polymerase chain reaction. RNA was harvested from VSMCs 24 hours after plating ($1x10^5$ cells/well, in quadruplicate), extracted using RNeasy minikit (Qiagen) following manufacturer’s instructions. 150 ng of RNA was reverse-transcribed (QuantiTect Rev. Transcription Kit; Qiagen) and 5 µl of cDNA (diluted 20-fold) was used to analyze the expression of *Atg5* using SYBR Green qPCR mix (Eurogentec) on a Roche lightcycler with the following primers: *Atg5* for 5’->3’: CCCCTGAAATGGCATTATCCAA; *Atg5* rev 5’->3’: AAAGTGAGCCTCAACCGCAT.
Il6 for 5’->3’: CCAGAGATACAAAGAAATGATGG; *Il6* rev 5’->3’: ACTCCAGAAGACCAGAGGAAAT. *Cxcl1* for 5’->3’: CAGACCATGGCTGGGATTCA; *Cxcl1* rev 5’->3’: AGTGTGGCTATGACTTCGGTTT. *Ccl2* for 5’->3’: GTTAACGCCCCACTCACCT; *Ccl2* rev 5’->3’: TTCTTTGGGACACCTGCTG. House keeping gene: *36b4* for 5’->3’: TCTCCAGAGGCACCATTGAAA; *36b4* rev 5’->3’ CTCGCTGGCTCCCACCTT.

Statistical analysis. Values are shown as average ± SEM. Statistical analysis was performed using Prism GraphPad and differences between groups were evaluated

using Mann-Whitney test (2 groups), Kruskal-Wallis test followed by uncorrected Dunn's test (> 2 groups), 2-way ANOVA (cell proliferation/survival) or Chi-squared test (Distribution between 2 groups), as indicated in the figure legends. Results were considered statistically different at $p<0.05$.

SUPPLEMENTAL FIGURES and FIGURE LEGENDS

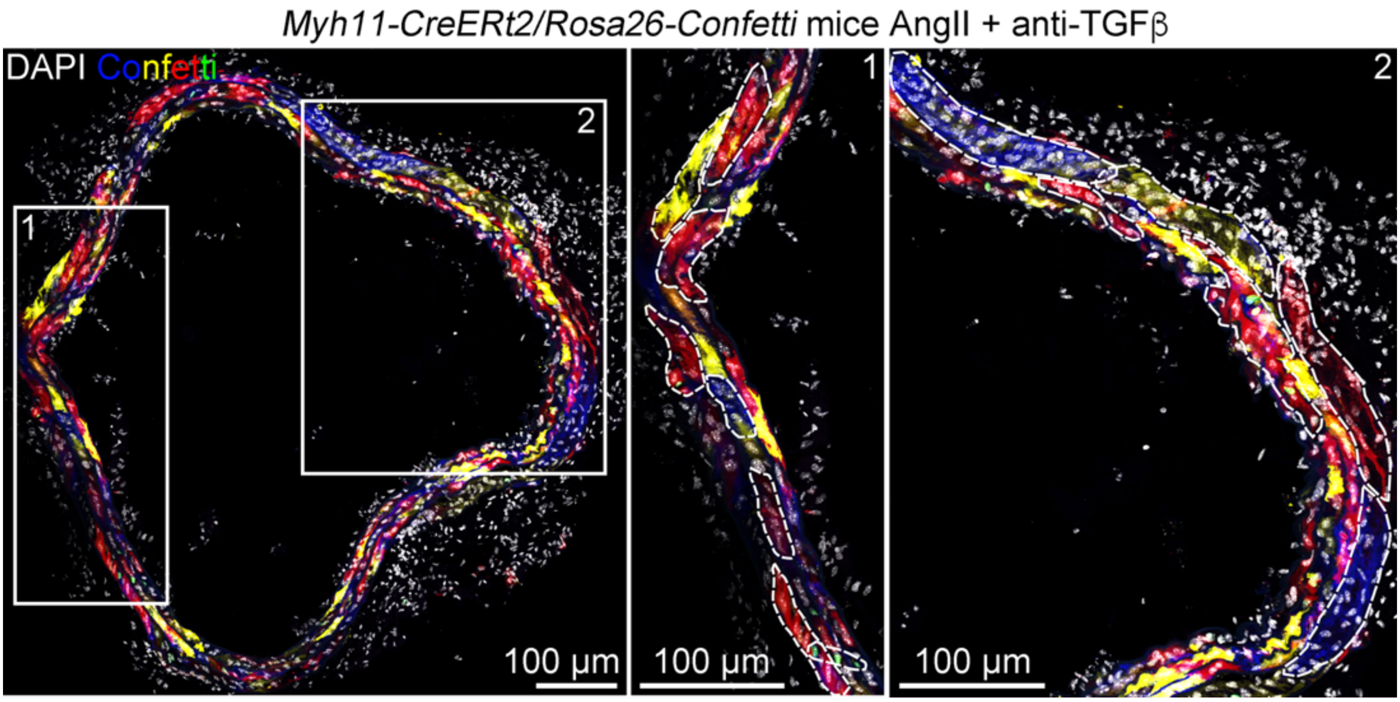

Figure I: Monochromatic patches in the media of *Myh11-CreERt2/Rosa26-Confetti* mice after AngII + anti-TGFβ infusion.
Representative pictures of monochromatic patches of VSMCs in the media of *Myh11-CreERt2/Rosa26-Confetti* mice after AngII + anti-TGFβ infusion analyzed by confocal microscopy. In the insets, dashed lines are circling monoclonal patches.

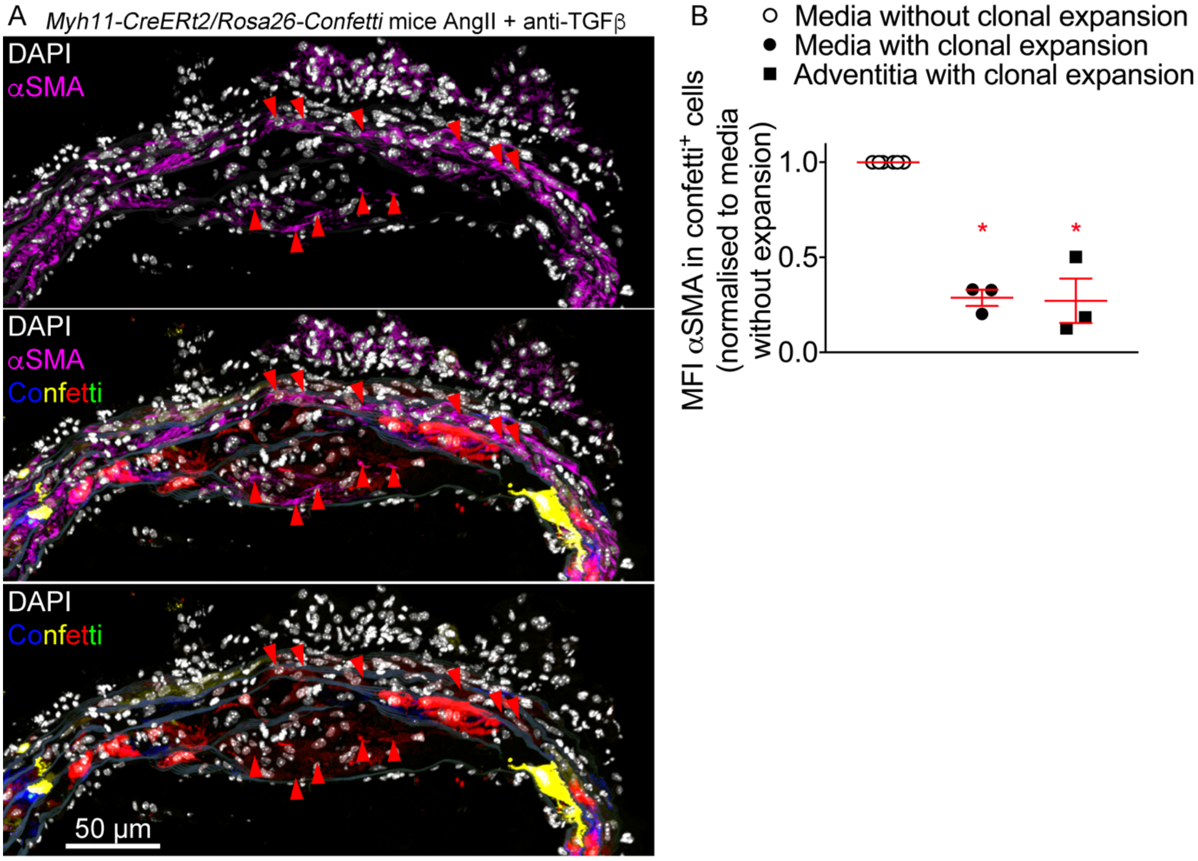

Figure II: Reduced expression of αSMA by clonally expanded medial VSMCs.
A – Representative images of αSMA staining of aortic cross sections from *Myh11-CreERt2/Rosa26-Confetti* mice after AngII + anti-TGFβ infusion. Monochromatic patches express lower levels of αSMA, compared to regions displaying the mosaic color pattern also observed in healthy vessels. Red arrow heads point to αSMA^+ Confetti-positive cells in a red patch of clonally expanded VSMCs.
B – Quantification of αSMA mean fluorescence intensity (MFI) in $Confetti^+$ cells from areas of the media, without clonal expansion (white circles) versus medial monochromatic patches (black circles, n=3) or adventitial outgrowths (black squares, n=3). For each region, 15 cells were analyzed per animal, and the MFI of αSMA from the expanded cells was normalized to the MFI of non-expanded cells from the same section (white circles). A total of 6 animals were used for quantification. *$p<0.05$ in media without clonal expansion vs other conditions. Kruskal-Wallis test followed by uncorrected Dunn's test.

Myh11-CreERt2/Rosa26-Confetti/Apoe$^{-/-}$ mice AngII + anti-TGFβ

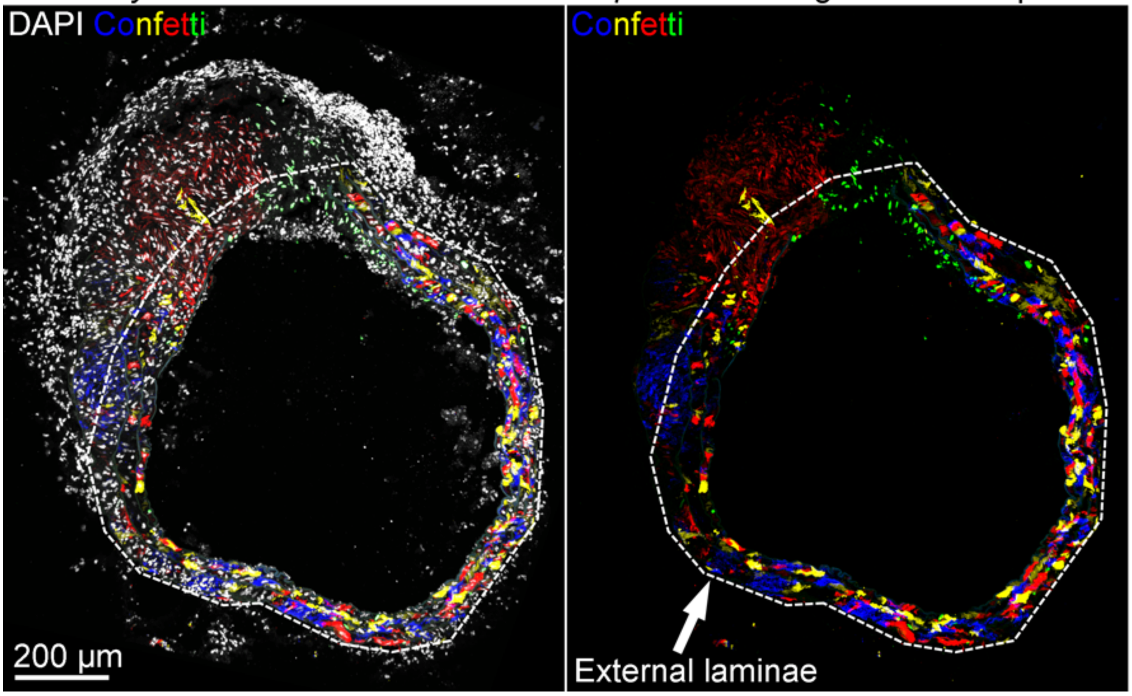

Figure III: Blocking TGFβ activity does not impair VSMCs outgrowth after aortic dissection.
Representative images showing clonally expanded VSMCs in the adventitia of a *Myh11-CreERt2/Rosa26-Confetti/Apoe*$^{-/-}$ animal that developed dissection after 28 days of AngII infusion and inhibition of TGFβ activity at day 14. The external elastic laminae is outlined.

Myh11-CreERt2/Rosa26-Confetti/Apoe$^{-/-}$ mice AngII

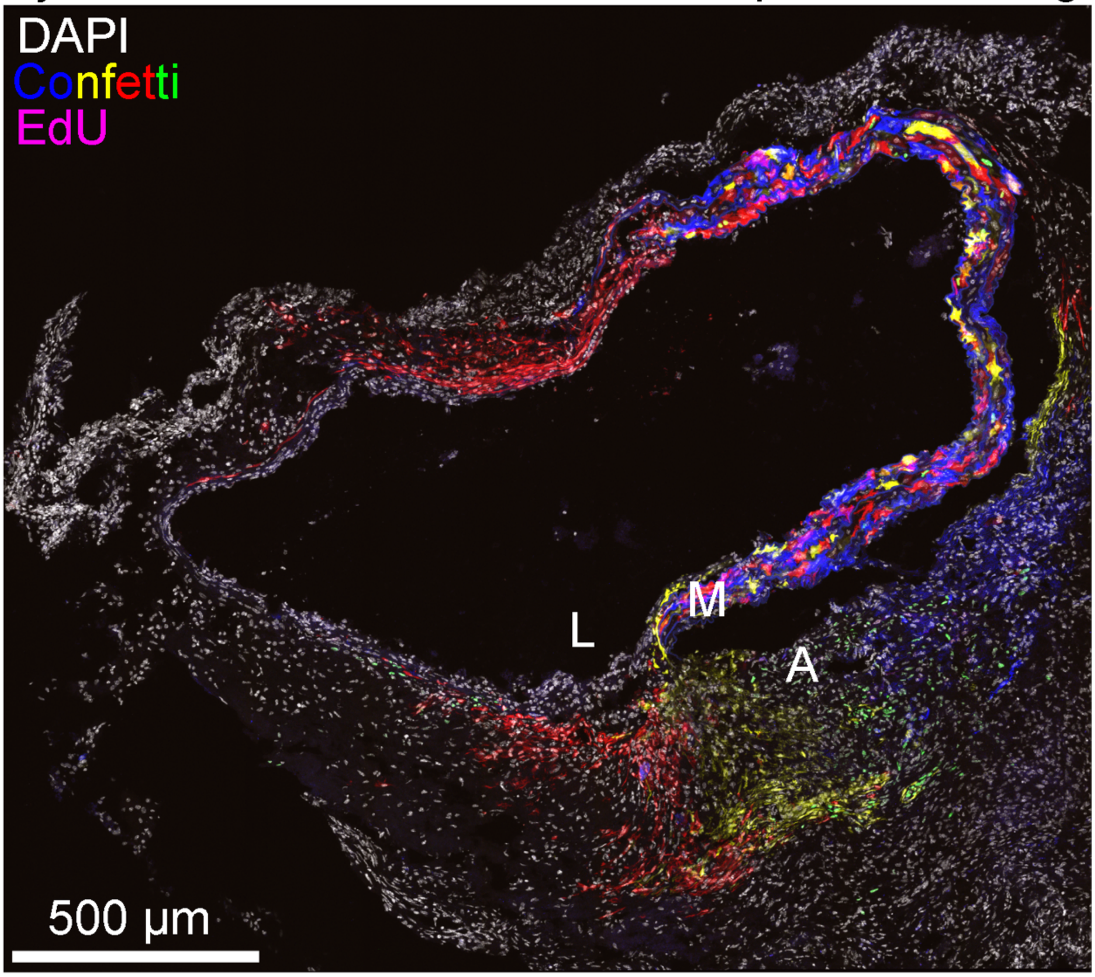

Figure IV: Representative image at low magnification of Figure 3F.
L: Lumen, M: Media, A: Adventitia.

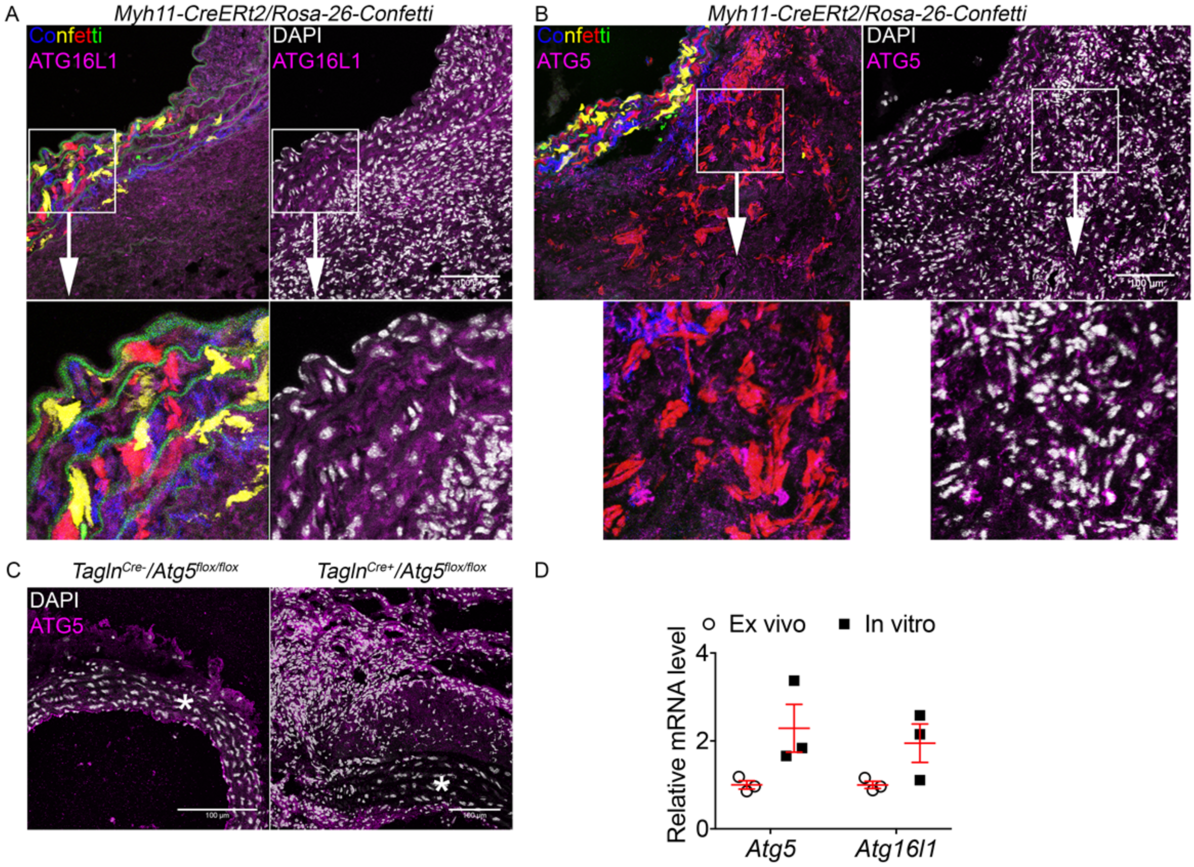

Figure V. Expression of autophagy related genes in phenotypically switched VSMCs.

A-C – Representative images of ATG16L1 (A) and ATG5 (B, C) staining (magenta) of aortic cross sections from AngII-treated, VSMC lineage labelled (*Myh11-CreERt2/Rosa26-Confetti)* mice with aortic dissection (A, B) and *Tagln*$^{Cre-,}$ *Atg5*$^{flox/flox}$ and *Tagln*$^{Cre+}$, *Atg5*$^{flox/flox}$ mice (C).

D – Analysis of *Atg5* and *Atg16l1* expression by RT-Q-PCR in *ex vivo* and primary VSMCs cultured at passage 4. Mean ± SEM of biological triplicates, normalized to house keeping genes (*Yhwaz* and *Hprt*) are shown.

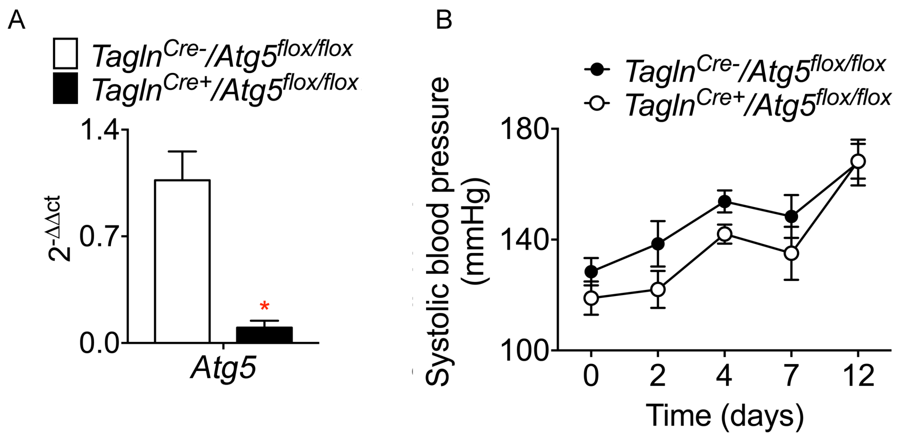

Figure VI. Expression of *Atg5* and blood pressure measurements in *Tagln*$^{Cre-}$ and *Tagln*$^{Cre+}$ *Atg5*$^{flox/flox}$ mice.

A – Analysis of *Atg5* expression by RT-Q-PCR of primary VSMCs derived from the aortas of *Tagln*$^{Cre-}$ and *Tagln*$^{Cre+}$ *Atg5*$^{flox/flox}$ mice. *$p<0.05$ *Tagln*$^{Cre-}$ vs *Tagln*$^{Cre+}$, Mann-Whitney test. Data were obtained using technical quadruplicates and are representative of two independent experiments.

B – Systolic blood pressure (mmHg) from *Tagln*$^{Cre-}$ (black, n=14 at the beginning of the experiment) and *Tagln*$^{Cre+}$ (white, n=17 at the beginning of the experiment) *Atg5*$^{flox/flox}$ mice before (day 0) and following AngII + anti-TGFβ infusion.

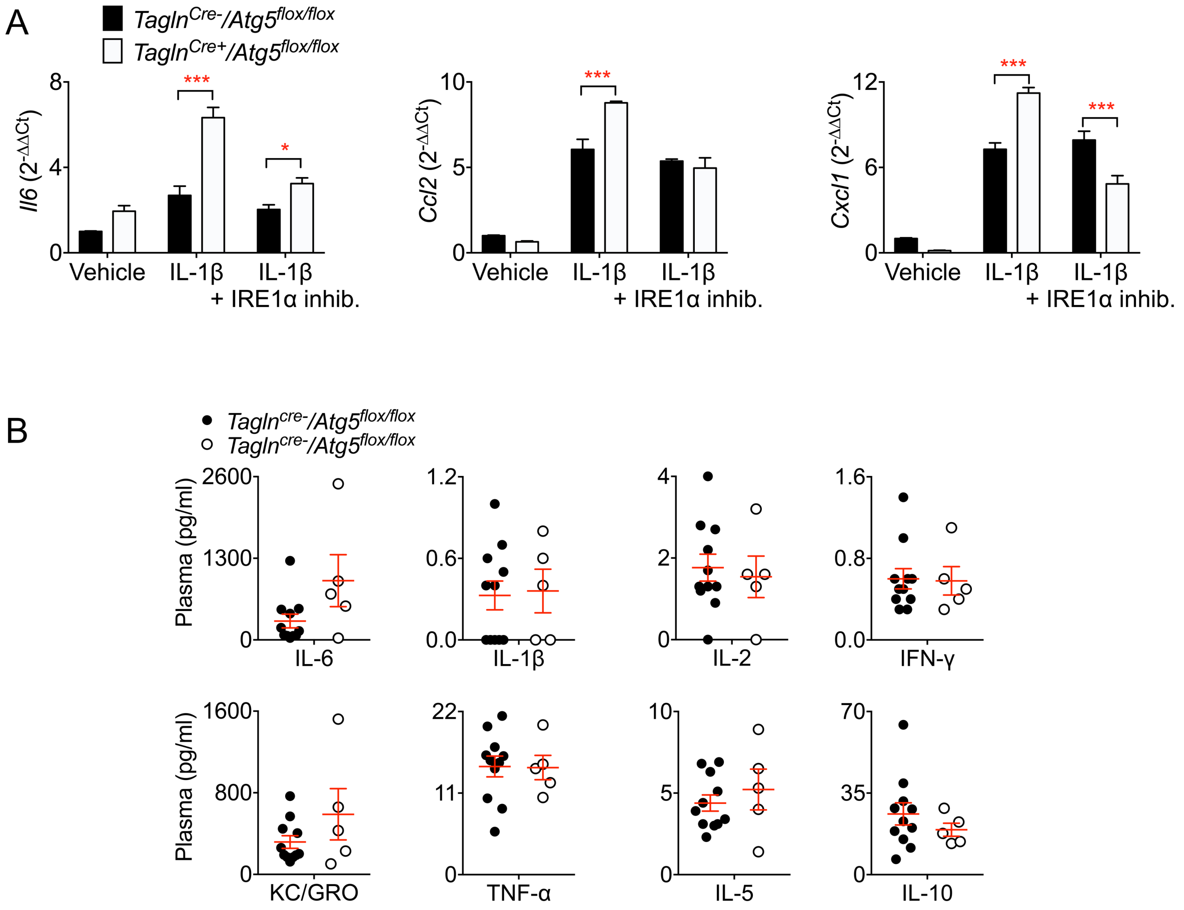

Figure VII. Atg5 deficiency in SMCs promotes a pro-inflammatory phenotype via IRE1α.

A- Analysis of *Il6*, *Ccl2* and *Cxcl1* expression by RT-Q-PCR of primary VSMCs derived from the aortas of *Tagln*$^{Cre-}$ + *Atg5*$^{flox/flox}$ and *Tagln*$^{Cre+}$ *Atg5*$^{flox/flox}$ mice stimulated for 16 hours with IL1β (1 µg/ml) in the presence or absence of the IRE1α kinase inhibitor (Apy29, 20µM) in vitro. Mean ± SEM of technical quadruplicates are shown. *p<0.05, ***p<0.001 *Tagln*$^{Cre-}$ vs *Tagln*$^{Cre+}$, 2-way ANOVA followed by uncorrected Fisher's test.

B- Analysis of circulating cytokines (IL-6, IL-1b, IL-2, IFNg, KC/GRO, TNFa, IL-5, IL-10) in the plasma of surviving *Tagln*$^{Cre-}$ *Atg5*$^{flox/flox}$ (n= 11) and *Tagln*$^{Cre+}$ *Atg5*$^{flox/flox}$ (n=5) mice infused with angiotensin II and anti-TGFβ at day 28.

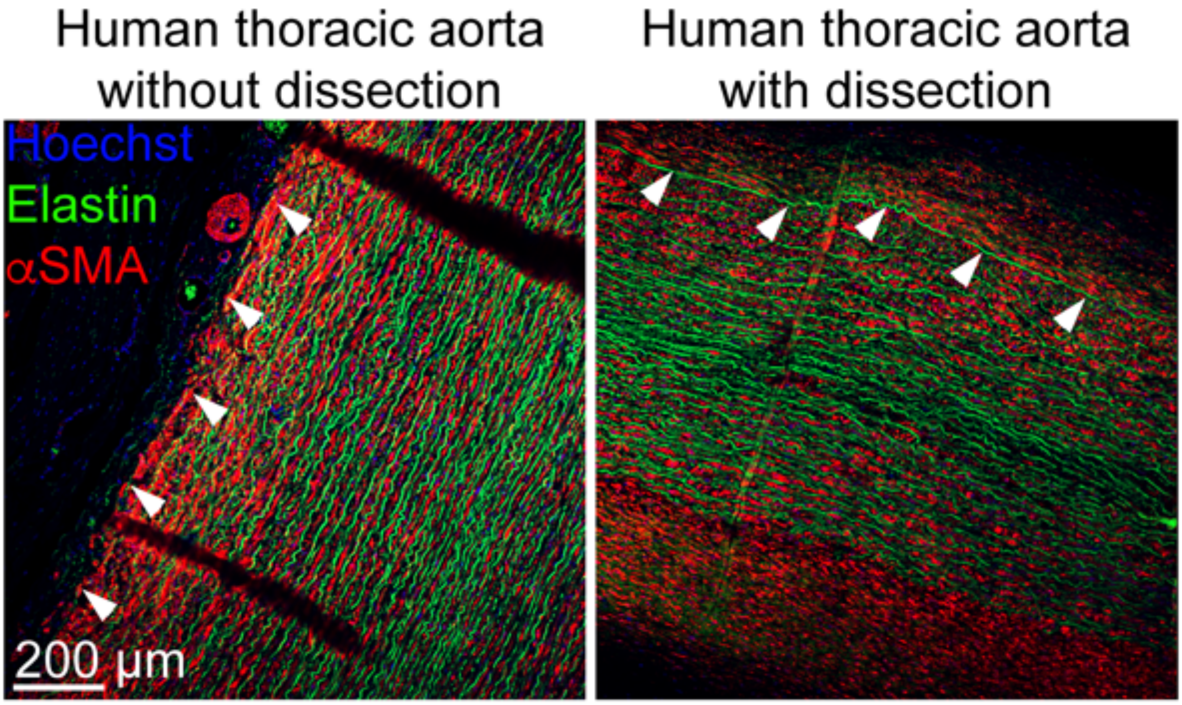

Figure VIII. αSMA expression in human thoracic aorta.
Representative images of αSMA staining on human thoracic aorta without dissection (n=5) or with dissection (n=5) showing αSMA^{+} cells in the adventitia of dissected aorta (arrow head showing the edge of the medial layer).

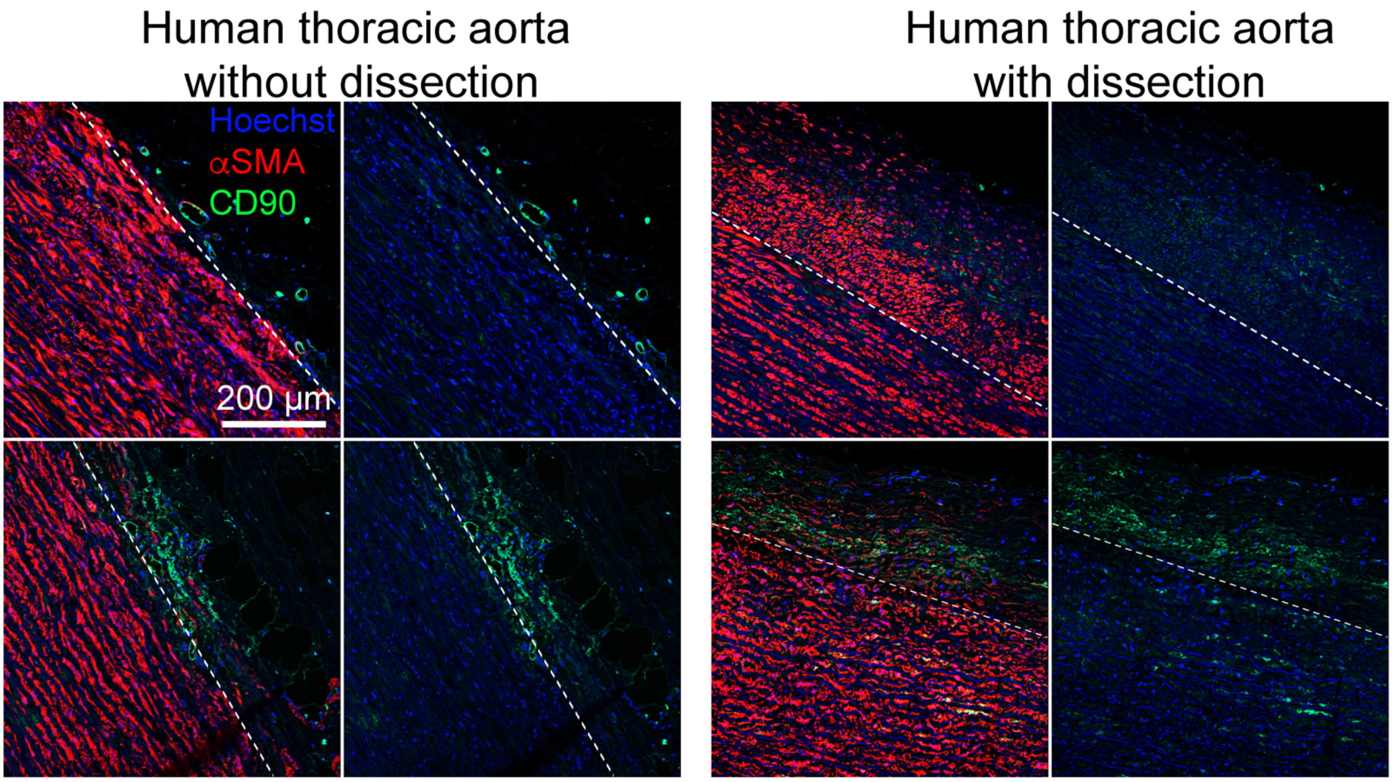

Figure IX. CD90^{-}αSMA^{+} Human SMCs accumulate to the adventitia after aortic dissection.
Representative examples of human thoracic aorta without dissections (2 examples are shown, 4 were analyzed) and with dissections (2 examples are shown, 5 were analyzed). Most of the αSMA^{+} cells outside of the media (limit shown by the dashed line) are CD90^{-}.

SUPPLEMENTAL REFERENCES

1. Chappell J, Harman JL, Narasimhan VM, Yu H, Foote K, Simons BD, Bennett MR and Jorgensen HF. Extensive Proliferation of a Subset of Differentiated, yet Plastic, Medial Vascular Smooth Muscle Cells Contributes to Neointimal Formation in Mouse Injury and Atherosclerosis Models. *Circ Res*. 2016;119:1313-1323.
2. Clement M, Haddad Y, Raffort J, Lareyre F, Newland SA, Master L, Harrison J, Ozsvar-Kozma M, Bruneval P, Binder CJ, Taleb S and Mallat Z. Deletion of IRF8 (Interferon Regulatory Factor 8)-Dependent Dendritic Cells Abrogates Proatherogenic Adaptive Immunity. *Circ Res*. 2018;122:813-820.
3. Hara T, Nakamura K, Matsui M, Yamamoto A, Nakahara Y, Suzuki-Migishima R, Yokoyama M, Mishima K, Saito I, Okano H and Mizushima N. Suppression of basal autophagy in neural cells causes neurodegenerative disease in mice. *Nature*. 2006;441:885-9.
