# Supplementary material for: Vascular Smooth Muscle Cell Plasticity and Autophagy in Dissecting Aortic Aneurysms
Source: Arterioscler Thromb Vasc Biol. 2019 Apr 4;39(6):1149–59. doi: 10.1161/ATVBAHA.118.311727 (PMC6544538; doi:10.1161/ATVBAHA.118.311727)
Supplement: Supplementary file 2 [file atv-39-1149-s002.pdf]

Major Resources Tables

Animals (in vivo studies)

Species	Vendor or Source	Background Strain	Sex
Mouse	Clevers lab (Snippert HJ et al., Cell 2010)	*Confetti*: Mixed background C56Bl/6 & 129P2/OlaHsd-derived IB10/E14IB10 embryonic stem (ES) cells	Female
Mouse	Offermans lab (Wirth A et al., Nat Med 2008)	*Myh11-CreERt2:* C57Bl/6	Male
Mouse	Charles River	*Apoe-/-*: C57Bl/6	Male
Mouse	In house breeding (experimental animals)	*Myh11-CreERt2/ROSA26/ Confetti*: Mixed background	Male
Mouse	In house breeding (experimental animals)	*Apoe-/-/Myh11-CreERt2/ROSA26/ Confetti*: Mixed background	Male
Mouse	Noburo Mizushima (Hara T et al., Nature 2006)	$Atg5^{flox/flox}$: C57Bl/6	Female
Mouse	Jax n°004746	$Tagln^{Cre+}$: C57Bl/6	Male
Mouse	In house breeding (experimental animals)	$Tagln^{Cre+}/Atg5^{flox/flox}$: C57Bl/6	Male

Animal breeding

	Species	Vendor or Source	Background Strain	Other Information
**Parent - Male**	Mouse	In house breeding	*Apoe-/-/Myh11-CreERt2/ROSA26/*	
**Parent - Female**	Mouse	Clevers lab (Snippert HJ et al., Cell 2010)	*Confetti*	

**Parent - Male**	Mouse	Jax n°004746	$Tagln^{Cre+}$	
**Parent - Female**	Mouse	Noburo Mizushima (Hara T et al., Nature 2006)	$Atg5^{flox/flox}$	

Antibodies

Target antigen	Vendor or Source	Catalog #	Working concentration	Lot # (preferred but not required)
αSMA	Sigma	C6198	5-10 µg/ml	
αSMA	Abcam	Ab125057	5-10 µg/ml	
HMOX1	Abcam	ab219360	5-10 µg/ml	
CD68	Biorad	MCA1957T	5-10 µg/ml	
LAMP2	SantaCruz biotech	sc-19991	5-10 µg/ml	
Atg16l1	Cell signaling technology	8089	5-10 µg/ml	
LC3	Cell signaling technology	3868	5-10 µg/ml	
Sqstm1/p62	Abcam	ab207305	5-10 µg/ml	
Active caspase 3	Cell signaling technology	9579	5-10 µg/ml	
IRE1α	Cell signaling technology	3294	5-10 µg/ml	
Ly6G	Thermofisher	14-5931-82	5-10 µg/ml	
GRP78/Bip	Cell signaling technology	3177	5-10 µg/ml	
TAGLN	Abcam	ab14106	5-10 µg/ml	
CD45	Thermofisher Biolegend	17-0451-82 103139	5-10 µg/ml	
CD31	Thermofisher	25-0311-82	5-10 µg/ml	
CD90.2	BDbiosciences	564365	5-10 µg/ml	
CD68	Biolegend	137021	5-10 µg/ml	
LAMP2	Thermofisher	11-1072-81	5-10 µg/ml	
Ter-119	Thermofisher	11-5921-85	5-10 µg/ml	

Cultured Cells

Name	Vendor or Source	Sex (F, M, or unknown)
WT and Atg5 KO SMCs	Primary culture	Females
