# Supplementary figures and images for: Vascular Smooth Muscle Cell Plasticity and Autophagy in Dissecting Aortic Aneurysms
Source: Arterioscler Thromb Vasc Biol. 2019 Apr 4;39(6):1149–59. doi: 10.1161/ATVBAHA.118.311727 (PMC6544538; doi:10.1161/ATVBAHA.118.311727)

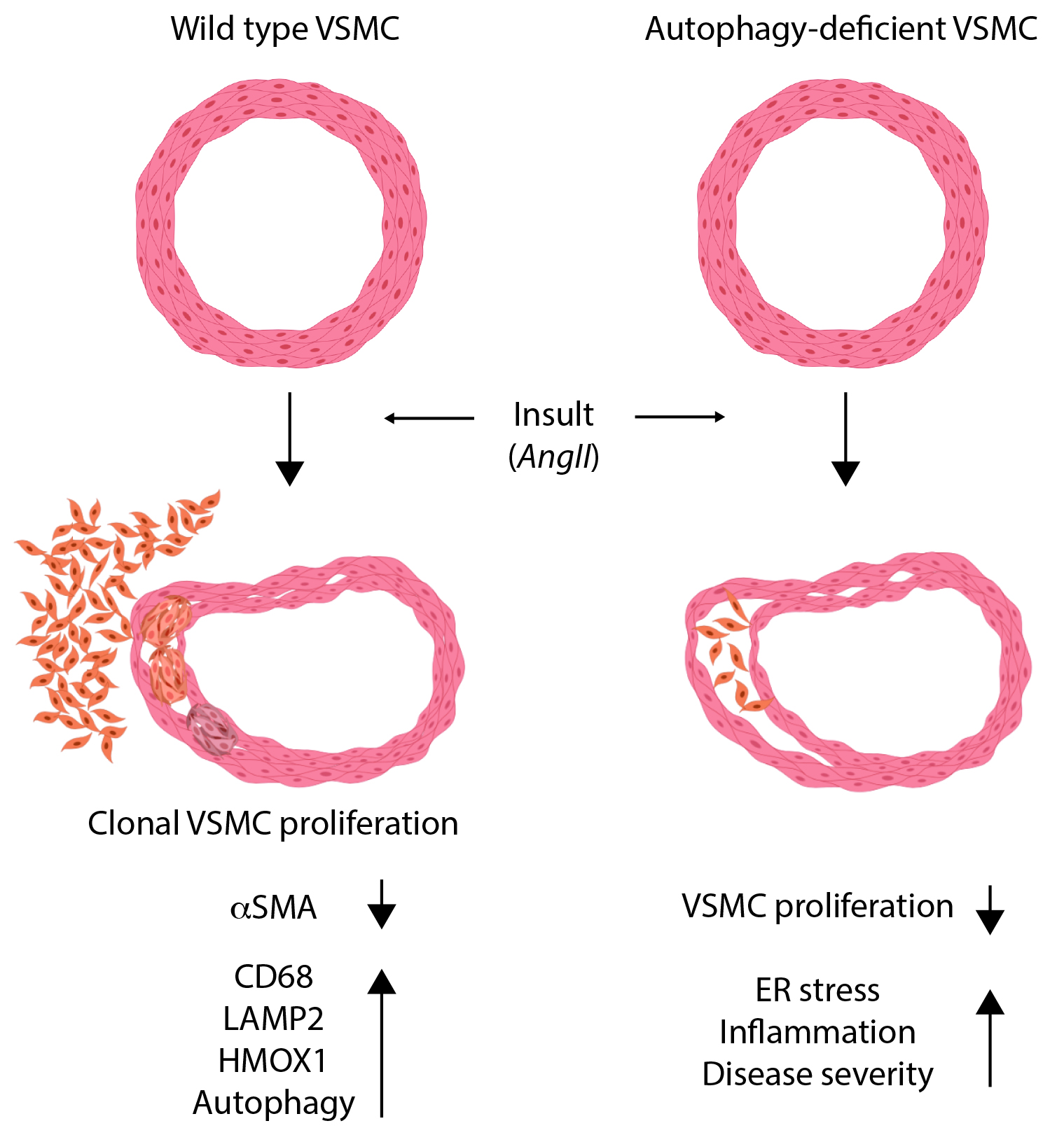

Supplement: Supplementary file 3 [file atv-39-1149-s003.jpg]
